# Fenton reaction mechanism generating no OH radicals in Nafion membrane decomposition

**DOI:** 10.1038/s41598-020-74646-0

**Published:** 2020-10-23

**Authors:** Takao Tsuneda

**Affiliations:** 1 Graduate School of Science, Technology, and Innovation, Kobe University, Kobe, 657-8501 Japan; 2 Department of Chemistry, Faculty of Science, Hokkaido University, Sapporo, 060-0810 Japan

**Keywords:** Chemistry, Energy science and technology, Nanoscience and technology

## Abstract

Mechanism of Fenton reaction, which is a most widely-used degradation test for organic materials using hydrogen peroxide (H$_2$O$_2$) and iron (Fe) cations, is revealed for the decomposition of hydrated Nafion membrane. This reaction mechanism has been assumed to generate OH radicals. For a doubly-hydrated Nafion membrane model, Fenton reaction with divalent and monovalent Fe (Fe$^{2+}$ and Fe$^+$) cation hydration complexes is explored for experimentally-supported hydration numbers using long-range correction for density functional theory. As a result, it is found that H$_2$O$_2$ coordinating to the Fe$^{2+}$ hydration complexes first approaches Nafion side chains in high humidity, then leads to the C–S bond dissociation of the side chain to produce carbonic acid group and sulfonic acid ion. On the other hand, once electron transfer proceeds between iron ions, the O–O bond of the coordinating H$_2$O$_2$ is extended, then the C–S bond is dissociated to produce trihydroxymethyl group and sulfur trioxide, which are rapidly transformed to carboxyl group and sulfonic acid ion in aquo. This mechanism is confirmed by the vibrational spectrum analysis of the decomposed product. Collective Nafion decomposition mechanisms also suggest that the decomposition reaction uses the recycle of generated Fe cation hydration complexes under acidic condition near membrane surface.

## Introduction

Radical-induced degradations of organic materials have been a critical problem in a wide range of fields of organic chemistry from molecular to biomedical sciences^1,2^. For many organic materials, the major cause for the degradations has been attributed to hydroxyl (OH) radicals, which are generated from the decomposition of hydrogen peroxide (H$_2$O$_2$). Various enhancement methods have so far been suggested: e.g., the introduction of phosphine oxide in proton exchange membranes of fuel cells^3^. In the durability test of organic molecules, Fenton reaction^4^ is frequently used, because this has been accepted to rapidly generate OH radicals decomposing organic molecules^5–7^. The Fenton reaction uses H$_2$O$_2$ solution with catalytic ferrous (Fe$^{2+}$) or other ions. This is a widely-used durability test for proton exchange membranes (PEMs) in fuel cells^8,9^. An infrared (IR) spectroscopic study^10^ shows that carbonic acid (–C(=O)OH) group is included in the dissolved species after the Fenton reaction of Nafion membrane. Fenton reaction mechanism of PEMS including Nafion has been explained as follows^11^:

1 $$\begin{aligned} \text{H}_2\text{O}_2 + \text{ Fe}^{2+}\rightarrow & {} \text{ OH } + \text{OH}^- + \text{Fe}^{3+}, \end{aligned}$$

2 $$\begin{aligned} \text{Rp-CF}_2\text{C(=O)OH } + \text{OH }\rightarrow & {} \text{ Rp-CF}_2 + \text{CO}_2 + \text{H}_2\text{O}, \end{aligned}$$

3 $$\begin{aligned} \text{ Rp-CF}_2 + \text{OH}\rightarrow & {} \text{ Rp-CF}_2\text{OH}, \end{aligned}$$

4 $$\begin{aligned} \text{ Rp-CF}_2\text{OH }\rightarrow & {} \text{ Rp-C(=O)F } + \text{ HF }, \end{aligned}$$

5 $$\begin{aligned} \text{ Rp-C(=O)F } + \text{ H}_2\text{O }\rightarrow & {} \text{ Rp-C(=O)OH } + \text{ HF }, \end{aligned}$$

where “Rp-CF$_2$C(=O)OH” represents Nafion membrane (“Rp” is the residual part of the Nafion) in their study, though it should be Rp-CF$_2$SO$_3$H before the Fenton reaction. Equation (1) follows the Haber–Weiss reaction mechanism mentioned later^12^. Note, however, that the lifetime of OH radicals is only 100 ns in aqueous solution, which is too short to cause degradation over a wide region in the proton conducting channels, where water clusters are present predominantly near the sulfonic acid groups. Actually, it has been questioned that the Fenton reaction of organic species contains OH radicals in aquo^13^. The author and coworkers also recently proposed that the decomposition of Nafion membrane proceeds by the direct reaction of H$_2$O$_2$ with the sulfonic acid groups without the production of OH radicals under the absence of metal ions^14^. On the other hand, seriously-different bond decomposition ratios are obtained for the decomposition of Nafion membrane between under the fuel cell operation condition and under the Fenton reaction^15^. The bond decomposition ratios are given approximately under a fuel cell operation condition (30 weight percent H$_2$O$_2$ at 80 $^\circ$C) as

6 $$\begin{aligned} \text{C}{-}\text{S} \text{ bond } \gg \text{C}{-}\text{F} \text{ bond }~~(7\% \text{ vs } 1\% \text{ after } \text{5.5 } \text{ days) }, \end{aligned}$$

and in the Fenton reaction as

7 $$\begin{aligned} \text{C}{-}\text{F} \text{ bond } > \text{C}{-}\text{S} \text{ bond }~~(68\% \text{ vs } 33\% \text{ after } 9\,\mathrm{h}). \end{aligned}$$

This result implies that as is different from the decomposition under the fuel cell operation condition, the Fenton reaction simultaneously decomposes two C–F and one C–S bonds of Nafion membrane.

The Fenton reaction mechanism has so far been discussed on the basis of the H$_2$O$_2$ decomposition reaction mechanism by Fe ions in many studies^9,16–18^. For the Fe-induced H$_2$O$_2$ decomposition, OH radicals have been taken as the main cause similar to the Fenton reaction. Haber and Weiss suggested a decomposition mechanism, which has been well-accepted as a mechanism for generating OH radicals^19^:

8 $$\begin{aligned} \text{ Fe}^{2+} + \text{ H}_2\text{O}_2\rightarrow & {} \text{ Fe}^{3+} + \text{ OH } + \text{ OH}^-, \end{aligned}$$

9 $$\begin{aligned} \text{ Fe}^{2+} + \text{ OH }\rightarrow & {} \text{ Fe}^{3+} + \text{ OH}^-, \end{aligned}$$

10 $$\begin{aligned} \text{ H}_2\text{O}_2 + \text{ OH }\rightarrow & {} \text{ HO}_2 + \text{ H}_2\text{O}, \end{aligned}$$

11 $$\begin{aligned} \text{HO}_2 + \text{H}_2\text{O}_2\rightarrow & {} \text{ O}_2 + \text{ H}_2\text{O} + \text{ OH }. \end{aligned}$$

As mentioned above, Eq. (1) of the Fenton reaction is based on Eq. (8) in the H$_2$O$_2$ decomposition reaction mechanism. Barb et al. modified this mechanism as^20,21^

12 $$\begin{aligned} 2\text{ Fe}^{2+} + 3\text{ H}_2\text{O}_2\rightarrow & {} ~2 \text{ Fe}^{3+} + 2\text{ OH}^-+ 2 \text{ H}_2\text{O } + \text{ O}_2. \end{aligned}$$

However, this OH-radical generation mechanism of Fenton reaction has been questioned in recent experimental studies. Bray and Gorin suggested the following mechanism around the same time as the Haber–Weiss one was suggested^22–24^:

13 $$\begin{aligned} \text{ Fe}^{2+} + \text{ H}_2\text{O}_2\rightarrow & {} \text{ FeO}^{2+} + \text{ H}_2\text{O}, \end{aligned}$$

14 $$\begin{aligned} \text{ FeO}^{2+} + \text{ H}_2\text{O}_2\rightarrow & {} \text{ Fe}^{2+} + \text{O}_2 + \text{ H}_2\text{O }. \end{aligned}$$

Based on an experimental kinetic analysis, Kremer reinterpreted the Haber–Weiss mechanism for avoiding OH-radical generation^25^: in an excess of ferrous ions over H$_2$O$_2$, ferrous ion is oxidized to ferric (Fe$^{3+}$) ion by H$_2$O$_2$,

15 $$\begin{aligned} 2\text{ Fe}^{2+} + \text{ H}_2\text{O}_2\rightarrow & {} ~2 \text{ Fe}^{3+} + 2\text{ OH}^-. \end{aligned}$$

In an excess of H$_2$O$_2$ over ferrous ions, H$_2$O$_2$ is decomposed accompanying this reaction,

16 $$\begin{aligned} 2\text{ H}_2\text{O}_2\rightarrow & {} ~2\text{ H}_2\text{O } + \text{ O}_2. \end{aligned}$$

Furthermore, the Fenton reaction rate is first given in the first order of both the concentration of ferrous ion and H$_2$O$_2$, but then, only the order of the ferrous ion concentration increases^20,21^. For the FeO$^{2+}$ production mentioned above, the following subsequent mechanisms are suggested to proceed besides the Bray–Gorin mechanism in Eq. (14)^25^:

17 $$\begin{aligned} \text{ FeO}^{2+} + \text{ Fe}^{2+} + \text{ H}_2\text{O}\rightarrow & {} \text{ 2Fe }^{3+} + \text{2OH }^-, \end{aligned}$$

18 $$\begin{aligned} \text{ FeO}^{2+} + \text{ Fe}^{3+}\rightarrow & {} \text{ FeOFe}^{5+} \end{aligned}$$

19 $$\begin{aligned} \text{ FeOFe}^{5+} + \text{ H}_2\text{O}_2\rightarrow & {} \text{ Fe}^{2+} + \text{ Fe}^{3+} + \text{O}_2 + \text{ H}_2\text{O }. \end{aligned}$$

The key of this mechanism is that FeOFe$^{5+}$ is produced in Eq. (18) for explaining the transient increase of the absorbance in real-time colorimetry at low H$_2$O$_2$ concentration. Recently, Enami and coworkers performed mass spectroscopy experiment of aqueous microjet containing Fe(II) chloride (FeCl$_2$) for selectively observing low-concentrated compounds produced at aqueous interfaces with very short time scale (< 5 $\times$ 10$^{-5}$ s) and found that the H$_2$O$_2$ decomposition proceeds not the OH radical formation but the FeO formation and it provides 1000–10,000 times larger reaction rate at the interface than that in aqueous solution^26^. Since this reaction mechanism, however, proceeds inside the hydration complex without the OH radical formation, it does not make clear how the Fe cation hydration complex decomposes organic materials such as Nafion membrane.

Very recently, the author and coworkers theoretically proposed a new H$_2$O$_2$ decomposition reaction mechanism based on this new experimental finding^27^: first, H$_2$O$_2$ is coordinated to the ferrous ion hydration complex, which takes long time to proceed at low H$_2$O$_2$ concentration and at requisite aqueous interface, and therefore explains the above-mentioned transient increase of the colorimetry absorbance^25^: the reaction is then driven by the electron transfer from ferrous cation hydration complexes to the H$_2$O$_2$-coordinating complex, i.e.,

20 $$\begin{aligned} ~[{\text{Fe}}^{{\mathrm{II}}}({\text{H}}_2\text{O})_6]^{2+}+ [{\text{Fe}}^{{\mathrm{II}}}({\text{H}}_2{\text{O}}_2)(\text{H}_2{\text{O}})_5]^{2+}\rightleftharpoons \,& [{\text{Fe}}^{{\mathrm{III}}}(\text{H}_2\text{O})_6]^{3+} + [{\text{Fe}}^{{\mathrm{I}}}(\text{H}_2\text{O}_2)(\text{H}_2\text{O})_5]^{+}. \end{aligned}$$

and then, the Fe$^+$ complex is rapidly oxidized to FeO hydration complex, i.e.,

21 $$\begin{aligned}~[{\text{Fe}}^{{\mathrm{I}}}({\text{H}}_2{\text{O}}_2)({\text{H}}_2{\text{O}})_5]^{+} \rightleftharpoons \, & [{\text{Fe}}^{{\mathrm{III}}} {{\text{O}}}({{\text{H}}}_2{{\text{O}}})_5]^{+} + {{\text{H}}}_2{{\text{O}}}, \end{aligned}$$

and finally, another H$_2$O$_2$ replaces with a coordinating H$_2$O of the FeO hydration complex to produce O$_2$ coordinating to the Fe cation hydration complex: i.e.,

22 $$\begin{aligned}&[{\text{Fe}}^{{\mathrm{III}}}{{\text{O}}}({{\text{H}}}_2{\text{O}}_2)({\text{H}}_2{\text{O}})_4]^{+} \rightleftharpoons [{\text{Fe}}^{{\mathrm{III}}}({\text{OH}})_2 ({\text{H}}_2{\text{O}}_2)({\text{H}}_2{\text{O}})_4]^{+} \\ &\qquad \rightleftharpoons [{\text{Fe}}^{{\mathrm{III}}} ({\text{OH}})({\text{HO}}_2)({\text{H}}_2{\text{O}})_4]^{+} \rightleftharpoons [{\text{Fe}}^{{\mathrm{I}}}({\text{H}}_2{\text{O}})_5{\text{O}}_2]^{+}.\end{aligned}$$

Note that the electronic structure of the produced O$_2$ molecule is transformed from the singlet to triplet states after a while. For Eqs. (21) and (22), we suggested another probable mechanism forming Fe(OH)$_2$ hydration complex, which bypasses the FeO formation: i.e., The Fe(OH)$_2$ hydration complex is first formed,

23 $$\begin{aligned} ~[{\text{Fe}}^{{\mathrm{I}}}({\text{H}}_2{\text{O}}_2)({\text{H}}_2{\text{O}})_5]^{+}\rightleftharpoons \, & [{\text{Fe}}^{{\mathrm{III}}}({\text{OH}})_2({{\text{H}}}_2{\text{O}})_4]^{+} + {\text{H}}_2{\text{O}},\end{aligned}$$

and then, O$_2$ is directly produced from the Fe(OH)$_2$ hydration complex coordinating H$_2$O$_2$,

24 $$\begin{aligned} ~[{\text{Fe}}^{{\mathrm{III}}}({\text{OH}})_2({\text{H}}_2{\text{O}}_2) ({\text{H}}_2{\text{O}})_4]^{+}\rightleftharpoons\,& [{\text{Fe}}^{{\mathrm{III}}} ({\text{OH}})({\text{HO}}_2)({\text{H}}_2{\text{O}})_4]^{+} \rightleftharpoons \, [{\text{Fe}}^{{\mathrm{I}}}({\text{H}}_2{\text{O}})_5{\text{O}}_2]^{+}.\end{aligned}$$

This latter mechanism is also consistent with the recent experimental result^26^, because it is known that Fe(OH)$_2$ is dehydrated to produce FeO.

Nafion membrane is the major proton exchange membrane in fuel cells. Since the decomposition rate of Nafion membrane mainly determines the durability of fuel cells, it has been frequently investigated in both experimental and theoretical studies^28–31^. For the decomposition of Nafion membrane, it is confirmed that the decomposition rate increases as the humidity decreases^28,29,32^, and H$_2$O$_2$ is generated by the reaction of hydrogen and oxygen on Pt surface^31^. Note that the decomposition of Nafion membrane proceeds even without metal ions^30^. Following these studies, the author and coworkers suggested a new direct H$_2$O$_2$-induced decomposition mechanism of Nafion membrane^14^. This mechanism is based on the hydration structure of Nafion membrane, which was revealed by the combination of experimental and theoretical studies^33,34^. That is, the IR spectroscopy and quantum chemistry calculation studies show that hydrated Nafion membrane has a double hydration structure of two sulfonic acid groups^33^, and the proton conductance in this membrane uses a relay mechanism through the doubly-hydrated structure under the low humidity condition^34^. Based on the doubly-hydrated structure, the author and coworkers theoretically explored the decomposition mechanism of Nafion membrane and consequently suggested a new decomposition mechanism of the ether-linkage by H$_2$O$_2$^14^. In this decomposition mechanism, H$_2$O$_2$ molecules hydrated in the double-hydration water cluster cleaves the ether-linkage of Nafion membrane, as experimentally confirmed^35–38^. Since as mentioned above, the decomposition of Nafion membrane under the fuel cell operation condition is found to be different from that in the Fenton reaction^15^, it is meaningful to explore how the Fenton reaction proceeds in the doubly-hydrated structures of Nafion membrane.

In this study, the decomposition mechanism of hydrated Nafion membrane in the Fenton reaction is theoretically investigated based on the above theoretical and experimental findings. In the calculations, the long-range correction (LC)^39,40^ for density functional theory (DFT)^41^ is used as one of the best tools for investigating electrochemical reactions. Based on our conventional studies on hydrated Nafion membrane^33,34^, the doubly-hydrated structure is examined as the calculation model of hydrated Nafion membrane. The Fenton reaction mechanism of Fe cation hydration complex, to which H$_2$O$_2$ molecule is coordinated, is revealed following our study on H$_2$O$_2$ decomposition^27^.

## Computational details

**Figure 1:**
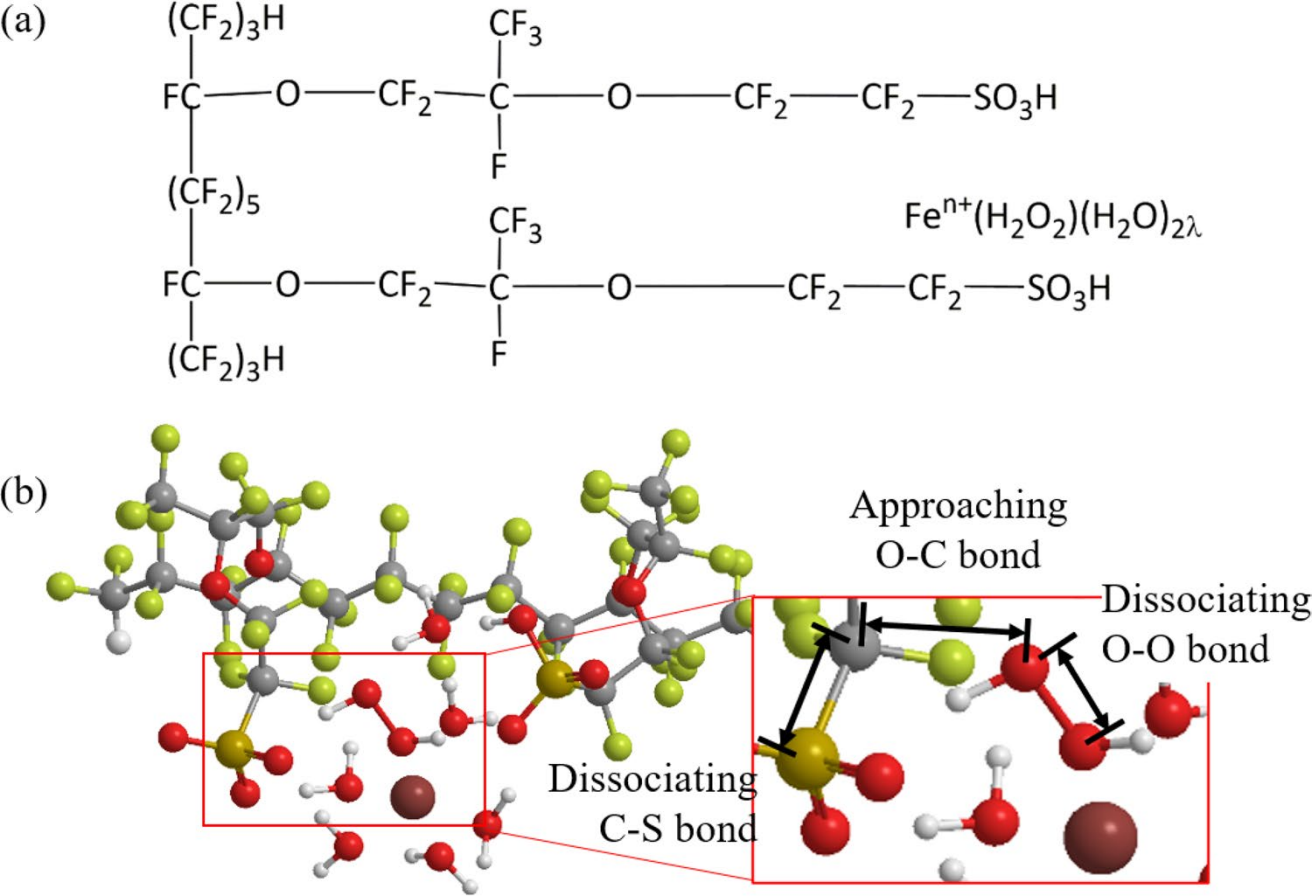
Calculation model of doubly-hydrated Nafion membrane plus Fe cation hydration complex coordinating H$_2$O$_2$: (**a**) chemical structure and (**b**) the optimized geometry at the hydration number per sulfonic acid group of $\lambda =3$. The approaching O–C bond and dissociating O–O and C–S bonds are also shown in (**b**) designed using ChemBio3D Ultra 12.0 (CambridgeSoft).

Based on our previous studies on the hydrated Nafion^33,34^, the double-unit model of hydrated Nafion membrane, which reproduces double hydration of the two sulfonic acid groups, has been adopted with the Fe cation hydration complex coordinating H$_2$O$_2$ molecule. This double hydration Nafion model has been confirmed both experimentally and theoretically, as mentioned above^33,34^. All calculations have been carried out using the LC^39,40^ for Becke 1988 exchange^42^ plus Lee–Yang–Parr correlation^43^ (LC-BLYP) functional (the only parameter $\mu$ = 0.33^44^) with the cc-pVDZ basis set^45,46^. Note that LC-DFT is the first-ever method quantitatively reproducing orbital energies and, therefore, can accurately reproduce reaction mechanisms driven by long-range electron transfers. Actually, LC-DFT has been repeatedly confirmed to correctly simulate various types of electrochemical reactions even including metal ions like iron ions^27,47^. Geometry optimizations of the hydrated Nafion plus Fe cation complex have been performed for several initial structures maximizing the number of the hydrogen bonds. In Fig. 1, the chemical structure and optimum geometries of the Nafion double-unit plus H$_2$O$_2$-coordinating hydrated Fe cation complex model are illustrated for the hydration numbers per sulfonic acid group: $\lambda =3$ (For other hydration numbers, see the supporting information). Using these optimum geometries as the initial structures, the geometries of the calculation models have been optimized by shortening the C–O distances between the closer O atom of H$_2$O$_2$ and the C atom of –CH$_2$SO$_3$ from those of the reactants to the products after making C–O bonds, and by extending the O–O and C–S bond distances at the values from 1.4 to 3.0 Å and from 1.8 to 6.0 Å, respectively, for the hydration numbers per sulfonic acid group $\lambda =3$, 4 and 5. The hydration numbers are based on the experimental findings in the actual fuel cell operation conditions showing that the hydration number per sulfonic acid group $\lambda$ is 3–5 for the relative humidity of 30–80% at 80 $^\circ C$^48^. These shortened and extended bonds are pointed in Fig. 1b, The Gaussian 09 suite of program^49^ has been used to perform all of the LC-BLYP calculations. All of the optimized structures have been checked to yield positive real frequencies. The GaussView 5.0.8^50^ has been used to analyze the vibrational modes contributing to IR spectra and their assignments.

## Calculated results and discussions

### H$_2$O$_2$ approach to a Nafion side chain

First, the approach of H$_2$O$_2$ molecule coordinating Fe cation hydration complex to the –CH$_2$SO$_3$ group of the side chain of Nafion is explored. The monovalent Fe cation (Fe$^+$) hydration complex besides the divalent (ferrous) one (Fe$^{2+}$) is examined, because it has been found in the previous study that H$_2$O$_2$ decomposition takes place by Fe$^+$ cation hydration complex, which is generated by the electron transfer from other Fe ions through hydrogen bond networks^27^.

**Figure 2:**
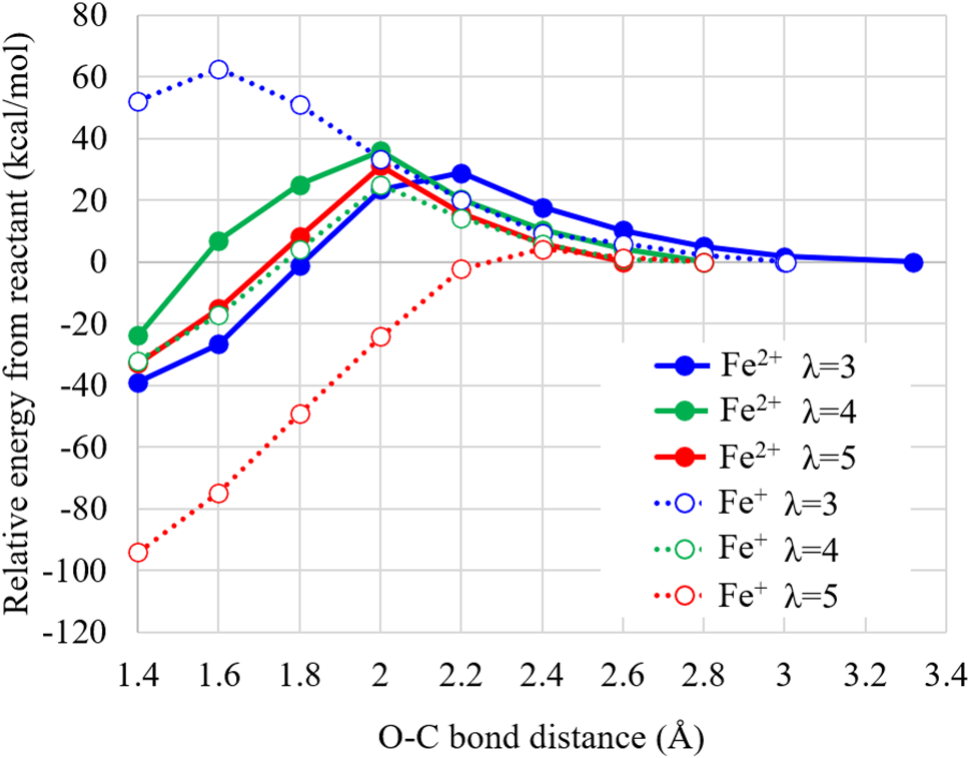
Potential energy curves of doubly-hydrated Nafion membrane model plus Fe cation hydration complex coordinating H$_2$O$_2$ with respect to the distance between the closer O atom of H$_2$O$_2$ and the C atom of the –CH$_2$SO$_3$ group of a Nafion side chain. The geometries are optimized with keeping the O–C bond distance at the coordinate point values. Zero potential energy is set as the total energy of the reactant for Fe$^{2+}$ and Fe$^+$ ion hydration complexes and for the hydration number of $\lambda$ = 3, 4 and 5. The hydration number indicates the number of included water molecules per the sulfonic acid groups (i.e., two groups are included in this model). LC-BLYP/cc-pVDZ/LANL2DZ is used.

Calculated potential energy curves (PECs) of the H$_2$O$_2$ approach are illustrated in Fig. 2. The figure shows that the reaction barriers have about 30 kcal/mol for ferrous ion hydration complex (28.8, 36.0 and 31.2 kcal/mol at the tops for $\lambda$ = 3, 4 and 5, respectively). Note that these barrier energies are close to the experimental activation energies of H$_2$O$_2$ decomposition and F$^-$ generation in the presence of Nafion film (20.3 and 23.2 kcal/mol, respectively), which are estimated using a simple first-order kinetic model^51^. This result, therefore, suggests that the bottleneck process of the Fenton reaction is the approach of H$_2$O$_2$ to the side chain for the ferrous ion hydration complex. In contrast, as shown in the figure, the reaction significantly depends on the hydration number for Fe$^{+}$ ion hydration complex: the reaction barriers dramatically decrease from 62.6 kcal/mol ($\lambda$ = 3) to 4.1 kcal/mol ($\lambda$ = 5) and the reaction energies considerably increase from − 52.1 kcal/mol ($\lambda$ = 3) to 94.1 kcal/mol ($\lambda$ = 5). This result seems to indicate that the Fenton reaction has high reactivity in high humidity after accepting electron. Note, however, that for the Fe$^{+}$ hydration complexes, the calculated reactants contain dissociated H$_2$O$_2$, which is OH coordinating the Fe$^{+}$ hydration complex and H$_2$O molecule after accepting proton. This is closely related to the H$_2$O$_2$ decomposition mechanism by iron ion hydration complexes under the absence of Nafion membrane^27^, as mentioned later in “Fenton reaction mechanism and collective Nafion decomposition mechanisms” section. In summary, the results show that the Fenton reaction proceeds with the barrier of about 30 kcal/mol for the ferrous ion hydration complex, while it significantly depends on the hydration number and requires the initial O–O bond extension of H$_2$O$_2$ for the Fe$^{+}$ hydration complex.

**Figure 3:**
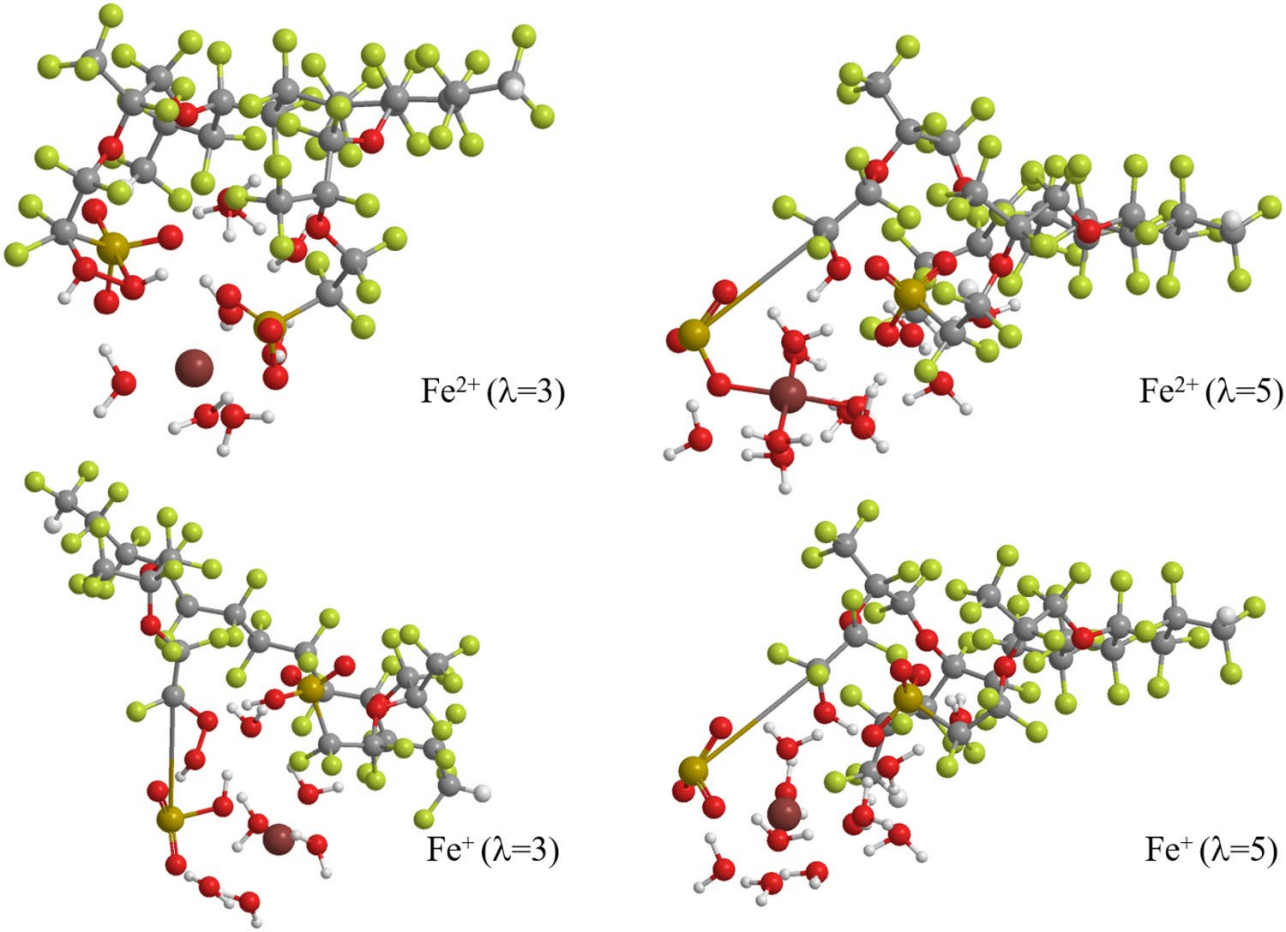
Optimum structures of doubly-hydrated Nafion membrane plus Fe cation hydration complex coordinating H$_2$O$_2$ after the H$_2$O$_2$ approach, for Fe$^{2+}$ and Fe$^+$ ion hydration complexes and for the hydration number $\lambda$ = 3 and 5. The hydration number indicates the number of included water molecules per the sulfonic acid groups (i.e., two groups are included in this model). LC-BLYP/cc-pVDZ/LANL2DZ is used. The structures are designed using ChemBio3D Ultra 12.0 (CambridgeSoft).

Figure 3 displays the optimized geometries of the reaction products, which are the H$_2$O$_2$-coordinating Fe cation hydration complex attaching the side chain of Nafion membrane after the H$_2$O$_2$ approach. As expected, the figure shows that the H$_2$O$_2$ approach makes the C–O bond with –CF$_2$SO$_3$ group of a Nafion side chain and leads to the C–S bond dissociation except for that of the ferrous ion complex for $\lambda$ = 3. This C–S bond dissociation is also found in the optimum structures of both ferrous and Fe$^+$ ion complexes for $\lambda$ = 4. Therefore, the H$_2$O$_2$ approach is closely related to the frequency of the C–S dissociation as described later in “C−S bond dissociation by Fe^2+^ hydration complex” section. The figure also indicates that H$_2$O$_2$ is dissociated to OH group forming –CF$_2$–OH and H$_2$O accepting proton for $\lambda$ = 5, while it is not dissociated to bond with the –CF$_2$ group for $\lambda$ = 3 (and also for $\lambda$ = 4 unillustrated), irrespective of the valency of the Fe hydration complexes. This result suggests that the hydration number significantly affects the product species probably due to the construction of hydrogen bond networks delivering protons in high humidity condition.

### C−S bond dissociation by Fe$^{2+}$ hydration complex

Let us focus on the C–S bond dissociation of a Nafion side chain, which simultaneously takes place with the H$_2$O$_2$ approach.

**Figure 4:**
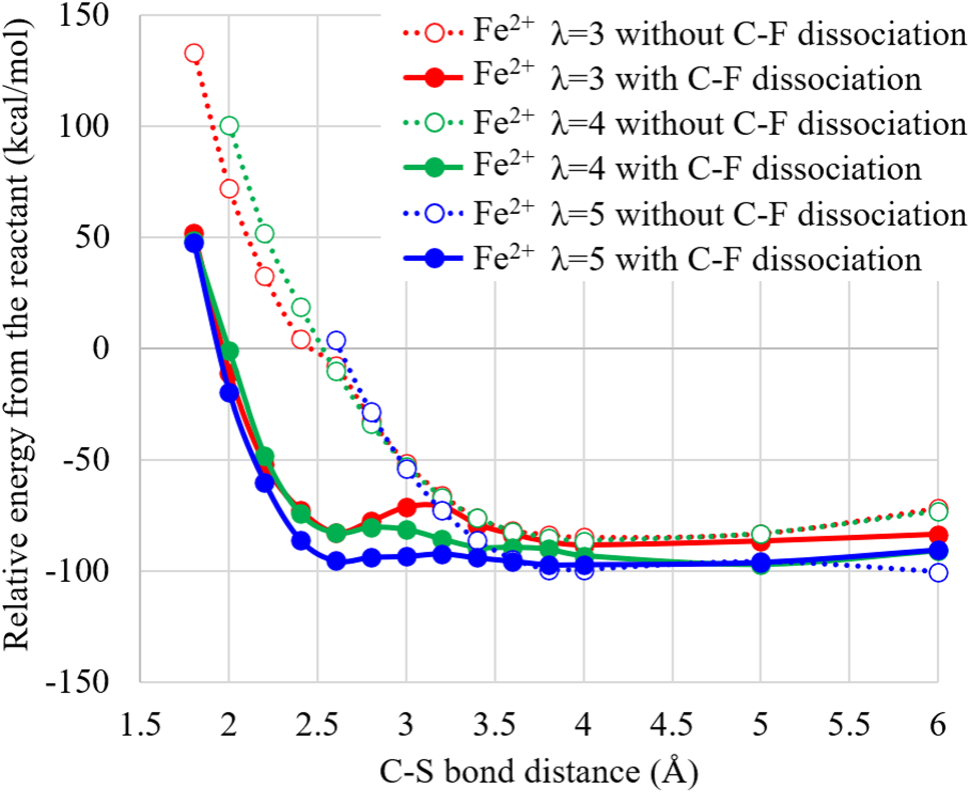
Potential energy curves of doubly-hydrated Nafion membrane model plus ferrous ion hydration complex coordinating H$_2$O$_2$ after the H$_2$O$_2$ approach with respect to the C–S bond distance of a Nafion side chain. The geometries are optimized for all the bonds but the C–S bond with keeping the C–S bond distance at the coordinate point values. Each zero potential energy is set as the total energy before the H$_2$O$_2$ approach, because all the curves provide no barriers for the C–S bond dissociation in, at least, the initial process. The $\lambda$ indicates the hydration number, which is the number of included water molecules per the sulfonic acid groups (two groups are included in this model). Note that several geometries are not obtained for the systems without the C–F dissociation, i.e., the system of $\lambda =4$ at the C–S distance of 1.6 Å and the systems of $\lambda =5$ at the C–S distances of 1.6, 1.8 and 2.0 Å, because they are inevitably optimized to the geometries with the C–F dissociation for these distances. LC-BLYP/cc-pVDZ/LANL2DZ is used.

Figure 4 plots the PECs of the Nafion membrane model plus the ferrous ion hydration complex with respect to the C–S bond distance of a Nafion side chain for the hydration numbers of $\lambda =3$, 4 and 5. The zero potential energy is set as the total energy of the reactant, which is the state before the H$_2$O$_2$ approach in “H_2_O_2_ approach to a Nafion side chain” section, for each hydration number. Note that the energies of the products are very similar despite of the different reactants dependent on hydration numbers. This result supports the high reliability of the calculated PECs. For the C–S dissociation, two types of the PECs are obtained for the accompaniment of the C–F bond dissociation of a Nafion side chain. That is, one of the C–F bonds next to the C–S bond spontaneously dissociates in a possible C–S bond dissociation, while it remains bonding in another possible C–S bond dissociation. The figure shows that the C–S bond dissociation has no barrier to take place for all situations but the $\lambda$ = 3 with the C–F bond dissociation. Comparing the PECs of the C–S dissociations with and without the C–F dissociation also shows that the C–F dissociation proceeds for the short C–S bond distances independent of the hydration numbers. Since this result indicates that the C–F bond dissociation takes place in the initial process of the Fenton reaction, it is consistent with the experimental finding that the C–F bond dissociation proceeds twice or more as much as the C–S bond dissociation in Fenton test^15^. These results clearly indicate that the C–S bond dissociation spontaneously proceeds with the C–F bond dissociations following the H$_2$O$_2$ approach in the presence of the ferrous ion hydration complex.

**Figure 5:**
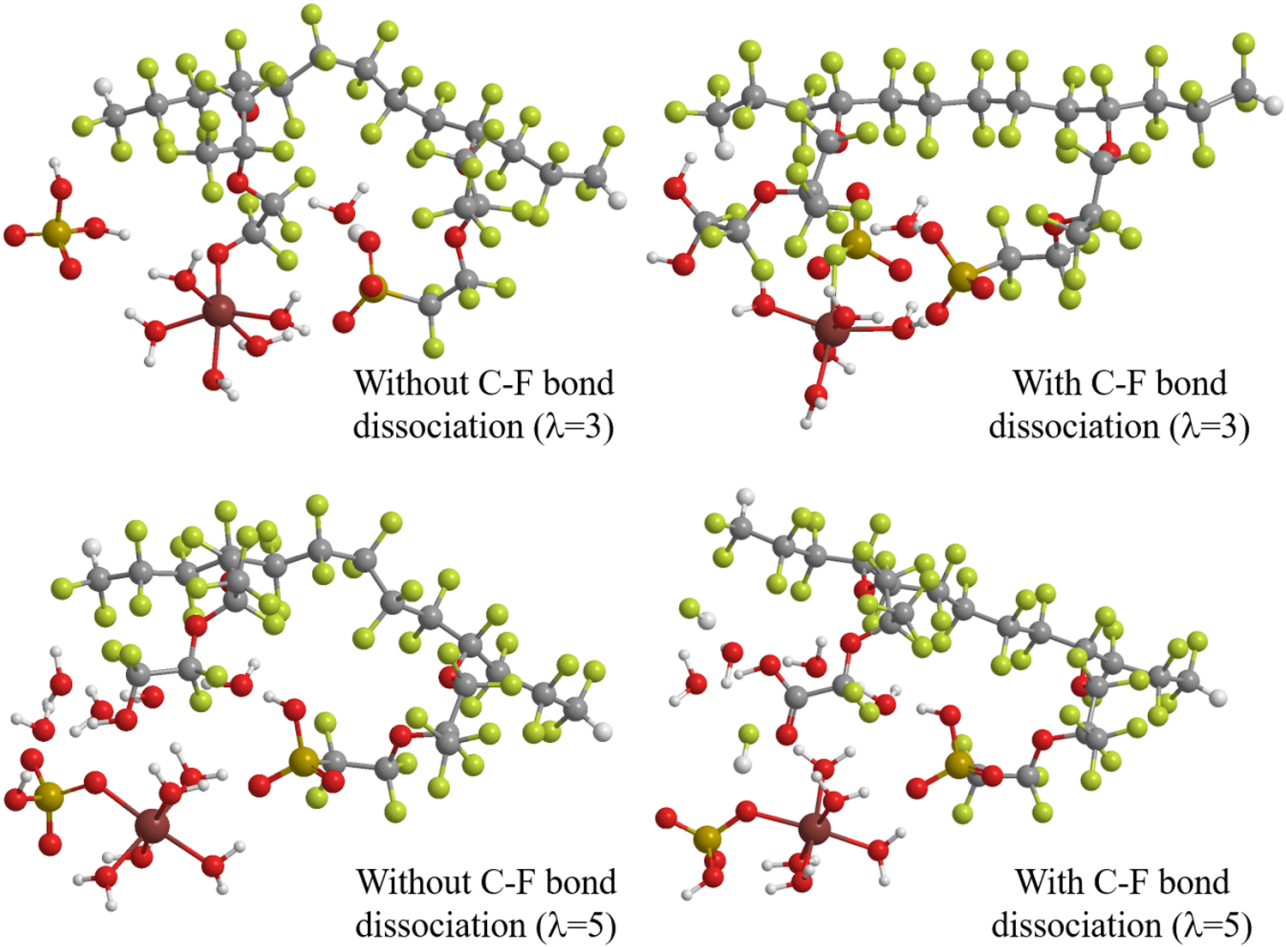
Optimum structures of doubly-hydrated Nafion membrane model plus ferrous ion hydration complex coordinating H$_2$O$_2$ for the geometrical change without and with the C–F bond dissociation of a Nafion side chain with keeping the C–S distance at 6.0 Å. LC-BLYP/cc-pVDZ/LANL2DZ is used. The structures are designed using ChemBio3D Ultra 12.0 (CambridgeSoft).

The optimized geometries of the products resulting from the C–S bond dissociation by the ferrous ion hydration complex are illustrated in Fig. 5. The figure shows that for $\lambda =3$ and 5, the C–S bond dissociation with the C–F bond dissociation produces the –C(OH)$_2$F and –C(=O)OH groups, in which one and two fluorine groups are dissociated, while the C–S bond dissociation without the C–F bond dissociation gives –CF$_2$O and –CF$_2$OH groups, respectively. Considering the PECs in Fig. 4, the C–S bond dissociation by the ferrous ion hydration complex spontaneously proceeds with the C–F bond dissociation for $\lambda =5$. It is, therefore, concluded that the ferrous ion hydration complex decomposes Nafion and produces a –C(=O)OH group by dissociating two C–F bonds. This conclusion is consistent with the experimental bond decomposition ratios, in which the C–F bond dissociation ratios are shown to be twice as the C–S bond ones in Fenton reaction for 9 h^15^.

For the C–S bond dissociation, note that this bond already dissociates during the H$_2$O$_2$ approach except for the coordination to the ferrous ion hydration complex of $\lambda$ = 3, as mentioned above. The correspondence of the results of the C–S bond dissociation with the experimental result indicates that the C–S bond dissociation simultaneously takes place from the H$_2$O$_2$ approach. Note, however, that the minimum total energies of the C–S bond dissociation are lower than those for the H$_2$O$_2$ approach. The optimum geometries suggest that this mainly comes from the difference in the stabilities of the product species: e.g., –CF$_2$ and –C(=O)OH groups and SO$_3$ and HSO$_4^-$ fragments for the H$_2$O$_2$ approach and the C–S bond dissociation of $\lambda$ = 5, respectively. Considering the barrier-less PECs of the C–S bond dissociation, it is, therefore, suggested that the H$_2$O$_2$ approach induces the C–S bond dissociation of Nafion membrane and provides carbonic acid –C(=O)OH group and sulfonic acid HSO$_4^-$ in the presence of the ferrous ion hydration complex.

### C−S bond dissociation by Fe$^+$ hydration complex

The Fe$^+$ hydration complex also induces the C–S bond dissociation of Nafion side chain in the H$_2$O$_2$ approach with a comparable reaction barrier to those of the ferrous ion complex for, at least, $\lambda$ = 4, as mentioned in “H_2_O_2_ approach to a Nafion side chain” section. In addition, H$_2$O$_2$ decomposition in Fe$^{2+}$-containing aqueous solution is driven by the Fe$^+$ complex, which is generated by the electron transfer between ferrous ions, under the absence of decomposed materials like Nafion membrane^27^. We should, therefore, take the Fe$^+$ hydration complex into account in discussing the C–S bond dissociation.

**Figure 6:**
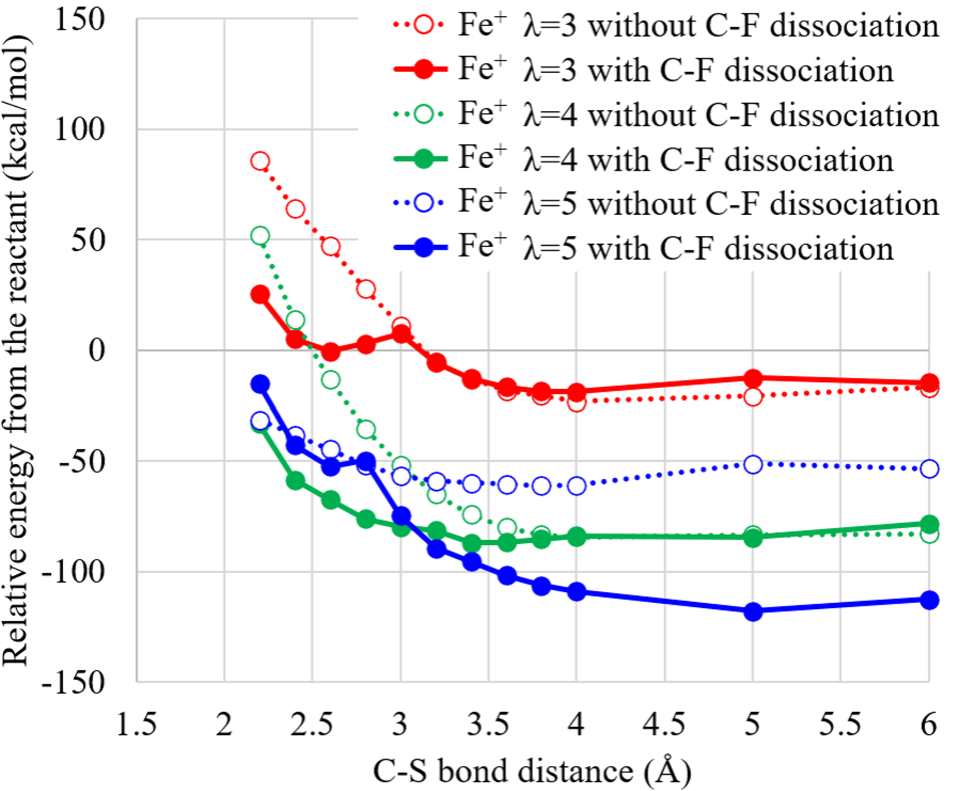
Potential energy curves of doubly-hydrated Nafion membrane model plus monovalent iron Fe(I) cation hydration complex coordinating H$_2$O$_2$ after the H$_2$O$_2$ approach with respect to the C–S bond distance of a Nafion side chain. The geometries are optimized for all the bonds but the C–S bond with keeping the C–S bond distance at the coordinate point values. Each zero potential energy is set as the total energy before the H$_2$O$_2$ approach, because all the curves provide no barriers for the C–S bond dissociation in, at least, the initial process. The $\lambda$ indicates the hydration number, which is the number of included water molecules per the sulfonic acid groups (two groups are included in this model). Note that the geometries with the C–F dissociation are not obtained at the C–S distance of 1.8 Å for all the hydration numbers, because they are inevitably optimized to the geometries without the C–F dissociation. LC-BLYP/cc-pVDZ/LANL2DZ is used.

Figure 6 displays the PECs in terms of the C–S bond dissociation by the Fe$^+$ hydration complex for three types of the hydration numbers. The figure shows that the PECs of the Fe$^{+}$ hydration complex significantly depend on the hydration numbers in contrast to the PECs of the Fe$^{2+}$ one. For the C–S bond dissociations with the C–F bond dissociations, the stabilization energies increase as the hydration number increases. This tendency is supposed to come from the hydration energy of hydrogen fluoride, which is formed by the C–S bond dissociation for all hydration numbers when the C–F dissociations are accompanied, as mentioned later. On the other hand, for the C–S bond dissociation without the C–F bond dissociations, the stabilization energies are $\lambda$ = 4, 5 and 3 in descending order. This may be due to the destabilization of the –CF$_2$OH group, which appears after the C–S dissociation when the C–F bond does not dissociate, in aqueous solution. These results indicate that the C–F bond dissociations spontaneously proceed with the C–S bond dissociations after H$_2$O$_2$ approach in high humidity condition in the presence of the Fe$^+$ hydration complex, similar to the reaction in the presence of the ferrous ion complex.

**Figure 7:**
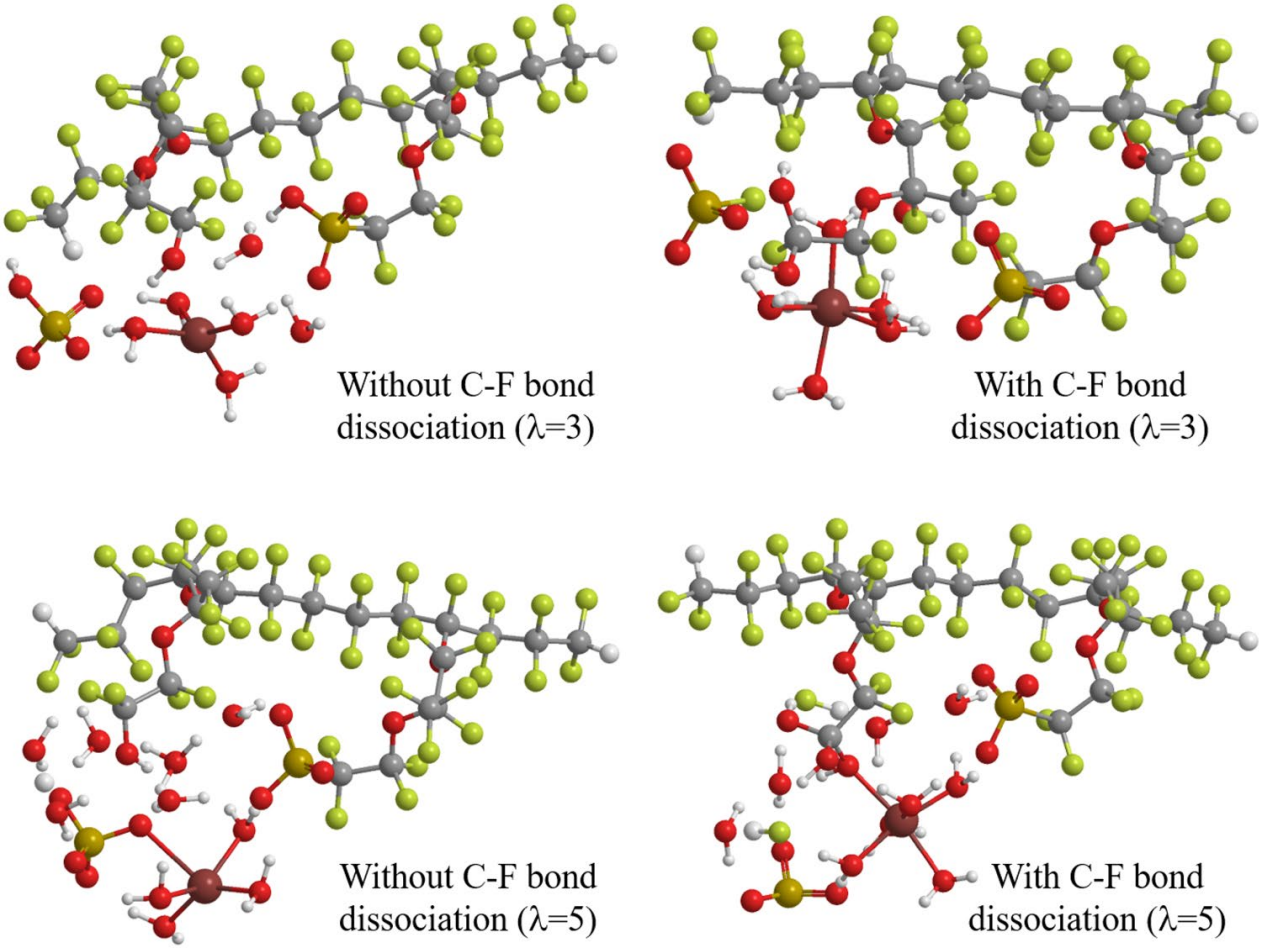
Optimum structures of doubly-hydrated Nafion membrane model plus Fe$^+$ hydration complex coordinating H$_2$O$_2$ for the geometrical change without and with the C–F bond dissociation of a Nafion side chain with keeping the C–S distance at 6.0 Å. LC-BLYP/cc-pVDZ/LANL2DZ is used. The structures are designed using ChemBio3D Ultra 12.0 (CambridgeSoft).

The optimized geometries of the products resulting from the C–S bond dissociation by the Fe$^+$ hydration complex are illustrated in Fig. 7. The figure shows that –C(OH)$_2$F and –C(OH)$_3$ groups are formed for $\lambda =3$ and 5 in the decompositions with the C–F bond dissociation, respectively, while –CF$_2$OH group is produced in the decompositions without the C–F bond dissociation independent of the hydration numbers. Since the number of produced hydrogen fluoride is different in the former case, this result supports that the significant hydration number dependence of the decomposition by the Fe$^+$ hydration complex comes from the stabilization of hydrogen fluoride in aqueous solution. It is also interesting to note the difference of the –C(OH)$_3$ (Fe$^+$) and –C(=O)OH (Fe$^{2+}$) groups of the products in high humidity ($\lambda =3$), because the same –C(OH)$_2$F group is formed in low humidity ($\lambda =3$). Moreover, the octahedral hydration structure of the Fe$^+$ is deformed except for the decomposition with the C–F bond dissociation for $\lambda =3$. For the accompaniment of the C–F dissociation, the C–S bond dissociation provides different decomposition fragments: SO$_3$F and SO$_3$ for $\lambda =3$ and $\lambda =5$, respectively. These differences may also contribute to the difference in the stability of the products. It is, therefore, suggested that the Fenton reaction process is clarified by carefully examining the decomposition products.

### Vibrational spectrum analyses of decomposed species after Fenton reaction

For confirming the decomposition products in the Fenton reaction, it is one of the most efficient ways to perform the vibrational spectrum analyses of the products by comparing them to the experimental IR spectrum.

**Figure 8:**
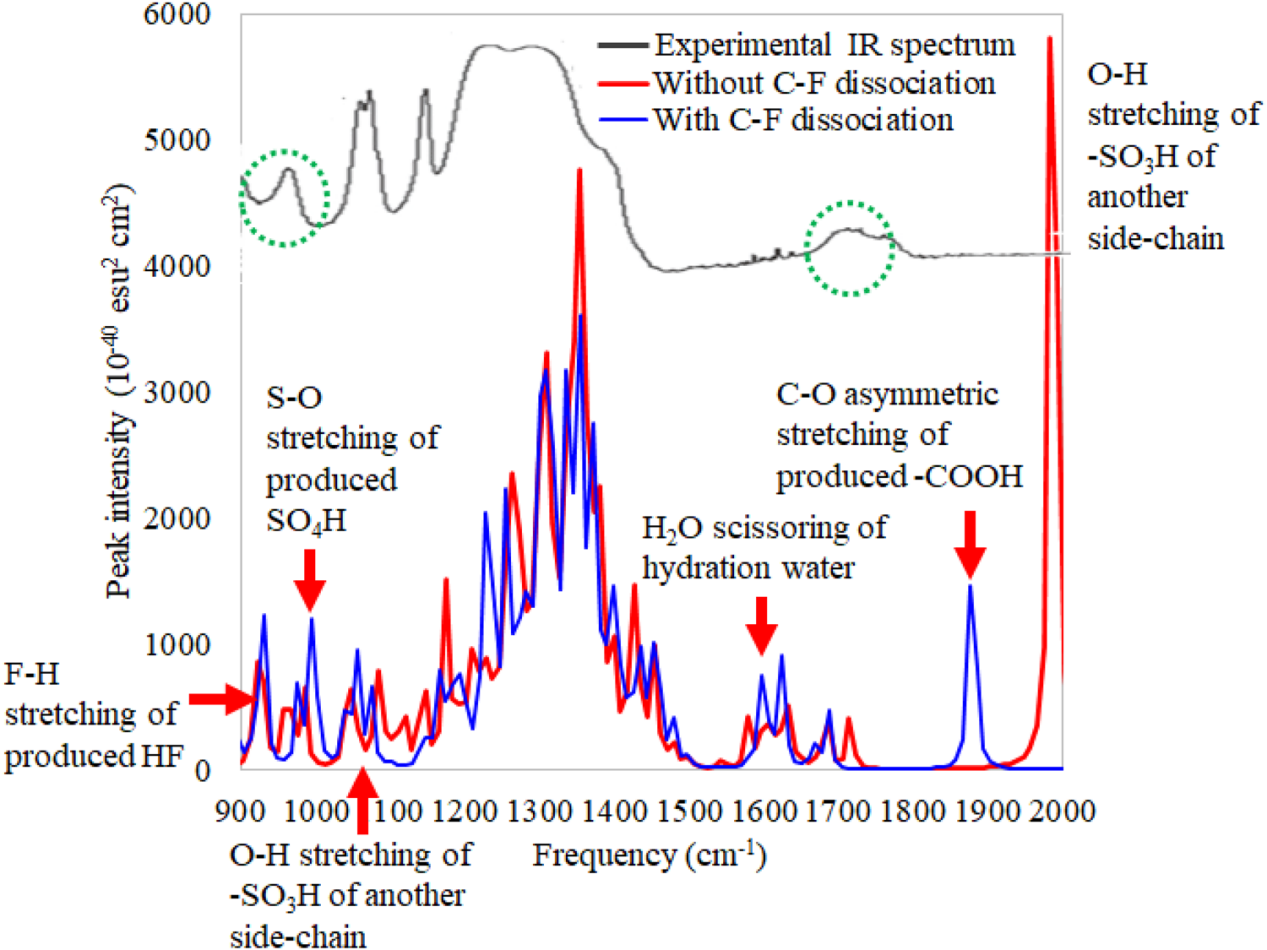
Vibrational spectra of doubly-hydrated Nafion membrane model with ferrous ion hydration complex after C–S bond dissociation with and without C–F bond dissociation for the hydration number of $\lambda =5$. For comparison, the experimental infrared spectrum of Nafion membrane after Fenton reaction in Ref.^10^, which is improved fitting to the calculated spectrum, is also shown. Newly-appeared peaks after the Fenton reaction are shown in green dotted circles. LC-BLYP/cc-pVDZ/LANL2DZ is used.

Figure 8 illustrates the calculated vibrational spectra of the Nafion model with the ferrous ion hydration complex resulting from the C–S bond dissociation (at the C–S distance of 6.0 Å) with and without C–F bond dissociation for $\lambda = 5$. The peak intensity is proportional to the dipole moment derivative for the normal vibrational mode. For comparison, the experimental IR spectrum^10^ is also shown in the region of 900–2000 cm$^{-1}$. The vibrational spectra of the products of $\lambda =5$ are compared to the experimental spectrum, because the experimental Fenton test is actually performed for 48 h under high humidity condition^10^. For each peak with frequency $\nu$, the Gaussian distribution function,

25 $$\begin{aligned} f(\nu )=\frac{1}{\sqrt{2 \pi \sigma ^2}}\exp \left[ -\frac{(\nu -\mu )^2}{2\sigma ^2}\right] , \end{aligned}$$

where $\sigma$ is the standard deviation ($\sigma =5$ is set in this study) and $\mu$ is the peak frequency, is multiplied. The figure shows that the calculated vibrational spectra well reproduce the IR spectrum of decomposed Nafion in the Fenton reaction, and that two newly-appeared peaks around 960 and 1730 cm$^{-1}$ correspond to the S–O stretching of the produced SO$_4$H and the C–O asymmetric stretching of the produced C(=O)OH group, respectively. Note that the very strong peak of the O–H stretching of -SO$_3$H group of the undecomposed side chain appears around 2000 cm$^{-1}$ only for the decomposition without the C–F bond dissociation. This is inconsistent with the experimental IR spectrum, in which no strong peak is given around 2000 cm$^{-1}$. This strongly supports our conclusion that the Fenton reaction simultaneously proceeds the C–S and C–F bond dissociations to give the –C(=O)OH group and SO$_4$H in, at least, long hours of the Fenton test.

### Fenton reaction mechanism and collective Nafion decomposition mechanisms

**Figure 9:**
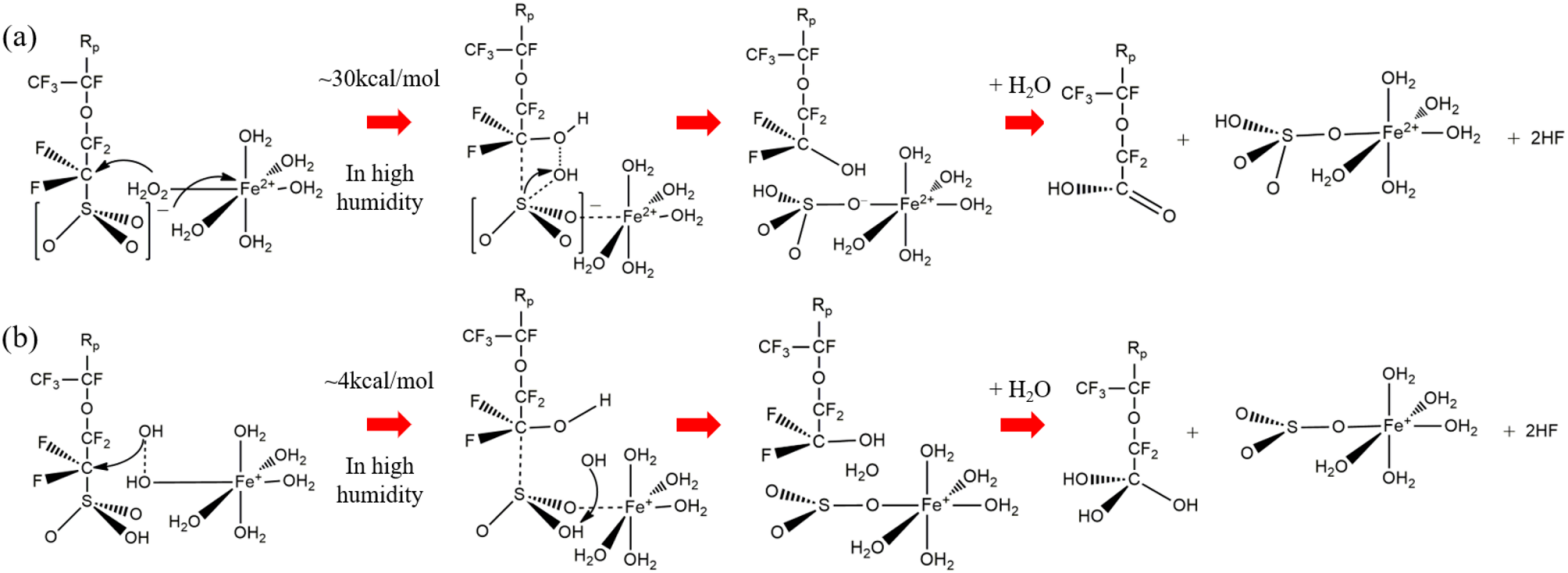
Schematic diagrams of Fenton reaction mechanism of hydrated Nafion membrane by Fe$^{2+}$ (**a**) and Fe$^+$ (**b**) cation hydration complexes.

Finally, let us focus on the Fenton reaction mechanism on the basis of the calculated results. The schematic diagram of the Fenton reaction mechanism for the ferrous ion hydration complex is drawn in Fig. 9a. For the Fe$^{2+}$ hydration complex, only the reaction mechanism of $\lambda$ = 5 is illustrated in this scheme, because it is either a most probable reaction process or the most compatible with the experimental conditions of Fenton test. As shown in the scheme, ferrous ion hydration complex makes coordinating H$_2$O$_2$ close to a side chain of Nafion, and forms the CF$_2$–OH and coordinating sulfonic acid. These products explain why it has been interpreted that Fenton reaction is induced by forming OH radicals, because they are the same as the presumable products of the OH radical-induced decomposition mechanism^11^. Then, the replacement of ligands and bond alternation takes place following the C–S bond dissociation to produce carbonic acids and Fe cation hydration complexes coordinating OSO$_3$H. Note that experimental Fenton tests have detected the peaks of the C(=O)OH group in IR spectra^10^. This C(=O)OH group has also been experimentally detected as a diacid with the SO$_3$H group in Nafion degradations^35–38^. The present reaction mechanism also produces the diacid by decomposing one C–S bond with keeping another C–S bond in the hydration region of doubly-hydrated Nafion. This suggests that the Fenton-like reaction also proceeds besides the direct H$_2$O$_2$-induced decomposition^14^ in Nafion decompositions when the hydration complexes of transition metal ions like ferrous or Pt$^{2+}$ ions being present near the side chain^52^. This reaction mechanism is consistent with all the experimental findings, as far as we know. However, the reaction barriers of about 30 kcal/mol, which is comparable to the barrier of Nafion degradation (30–40 kcal/mol)^14^, seem too high to explain the remarkably high Fenton reaction rate, though this rate is close to the experimental one as mentioned above^51^.

The Fenton reaction mechanism for the Fe$^+$ hydration complex in high humidity explains the cause for the discrepancy between the experimental high reaction barrier and high reaction rate. Figure 9b illustrates the schematic diagram of the Fenton reaction mechanism for the Fe$^{+}$ hydration complex. As shown in the figure, the reaction of the H$_2$O$_2$-coordinated Fe$^+$ hydration complex initially takes place from the O–O bond extension of H$_2$O$_2$ for $\lambda$ = 5. It is interesting to note that this O–O bond extension is confirmed to spontaneously proceed in H$_2$O$_2$-coordinating Fe$^{+}$ hydration complex^27^, while electron transfer between Fe cation hydration complexes is the bottleneck process. Though H$_2$O$_2$ decomposition by Fe cation hydration complexes also use this O–O bond extension as the initial process, this reaction may not have a significant effect on the Fenton reaction, because the former needs more energy (about 14 kcal/mol) than the latter one (about 4 kcal/mol) to proceed. The Fenton reaction using the Fe$^+$ hydration complex gives the trihydroxymethyl –C(OH)$_3$ group and sulfur trioxide SO$_3$ as the products, though none of them are experimentally observed. This discrepancy does not matter, because it is known that these species are immediately transformed into carbonic acid and sulfonic acid in aqueous solution, both of which are observed. Therefore, it is concluded that Fenton reaction initially proceeds by ferrous ion hydration complex with the barrier of about 30 kcal/mol, and then accelerated by electron transfer between Fe ions, this reaction rapidly progresses by Fe$^+$ hydration complex with the barrier of about 4 kcal/mol to produce carbonic acid group, sulfonic acid and hydrogen fluoride.

**Figure 10:**
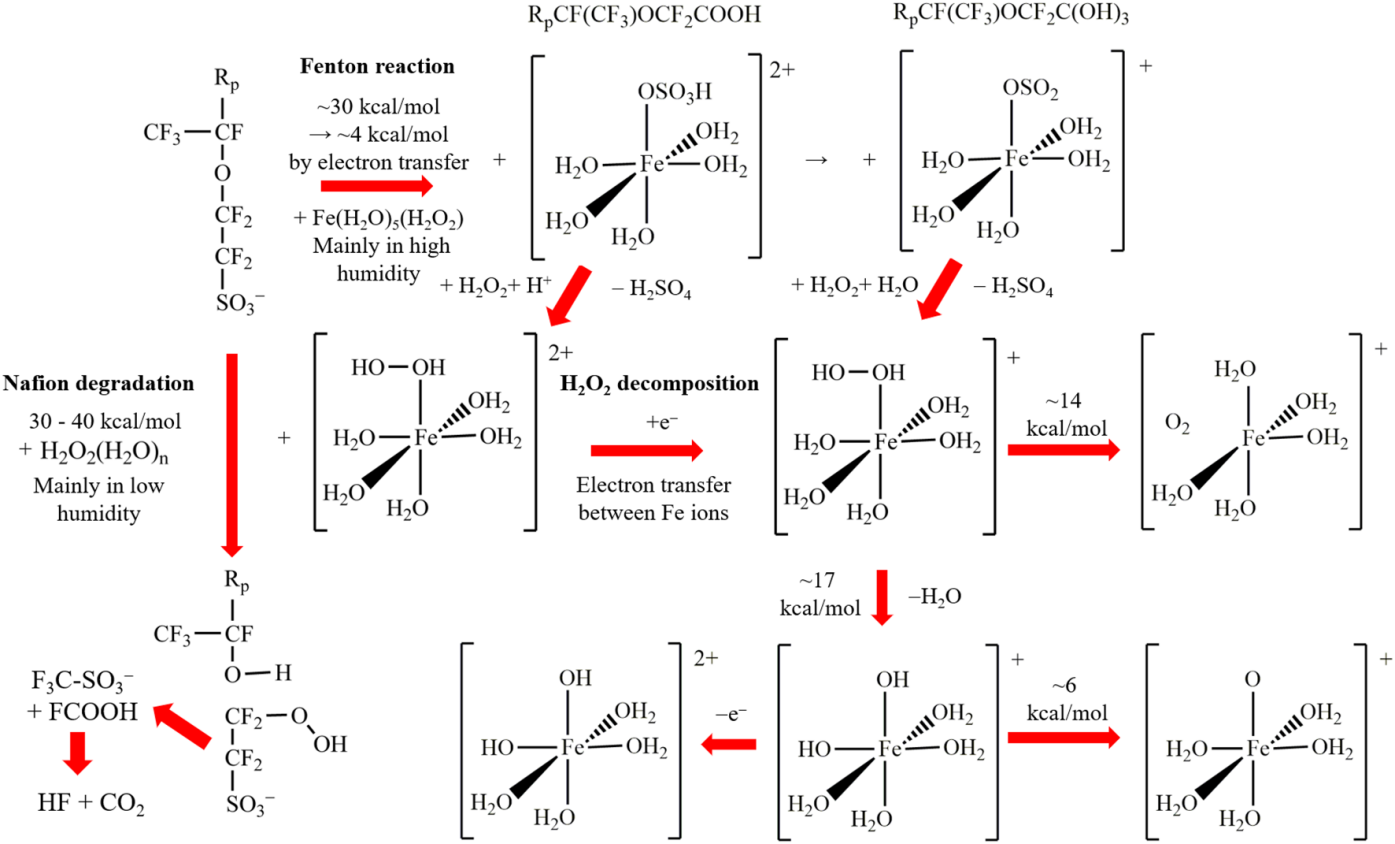
Schematic collective diagrams of hydrated Nafion membrane decomposition by H$_2$O$_2$ with and without Fe ions and subsequent reactions in high humidity.

For investigating the reaction mechanisms of the Nafion decomposition more comprehensively, it is also interesting to show the collective reaction mechanisms of Nafion membrane decompositions by H$_2$O$_2$ with no Fe cation hydration complexes^14^ and H$_2$O$_2$ decomposition^27^ besides the present Fenton reaction mechanism. Figure 10 combines the reaction mechanisms of the Nafion decomposition by H$_2$O$_2$ in the presence and absence of Fe cation hydration complexes with H$_2$O$_2$ decomposition. The figure shows that in the side chain, the ether C–O bond is decomposed in H$_2$O$_2$ aqueous solution with the reaction barrier of 30–40 kcal/mol, while the C–S bond is spontaneously split by H$_2$O$_2$ coordinating to the ferrous ion hydration complex in the Fenton reaction. This is consistent with conventional experimental findings. The reacting ferrous ion hydration complex of the Fenton reaction is the same as that of the H$_2$O$_2$ decomposition^27^. The H$_2$O$_2$ decomposition is, therefore, presumed to take place as a side reaction when no decomposed species being around this complex.

This collective Nafion decomposition mechanism also suggests that the generated Fe cation hydration complex replaces OSO$_3$H with H$_2$O$_2$ under acidic condition. This is because Nafion electrolyte membrane has very strong acidity and deteriorates near the interface with electrodes. There are a plenty of protons dissociating OSO$_3$H to produce stable H$_2$SO$_4$. The ligand replacement by H$_2$O$_2$ is also experimentally reported to proceed near the interface^26^. This suggests that generated Fe cation hydration complexes are recycled by the ligand H$_2$O$_2$ replacement as shown in Fig. 10. This suggestion explains the reason why the conversion of the –SO$_3$H group to the –SO$_3$M group (M=Na, Cs and Li) dramatically lowers the degradation rate^9^, because the latter provides much lower acidity than that of the former. This recycle is considered to promote the Nafion decomposition even with a very small amount of the Fe cation hydration complexes. This also indicates that the doubly-hydrated structure of Nafion membrane^33,34^, contributes to the decomposition, because it contains another undecomposed sulfonic acid group in the hydration region. These decomposition-accelerating factors may also contribute to the large difference between the reaction rates of the Fenton reaction and the main actual Nafion decomposition in fuel cells^15^.

## Conclusions

In this study, the mechanism of Fenton reaction^4^, which is frequently used in the degradation test of organic materials, has been theoretically investigated for the decomposition of hydrated Nafion membrane. The reactions of a doubly-hydrated Nafion membrane model^33,34^ with Fe cation hydration complexes are explored for three types of hydration numbers (the hydration number per sulfonic acid group $\lambda =3$, 4 and 5) using the long-range correction for density functional theory and that is the only functional quantitatively reproducing electron transfers^39–41^. Consequently, a composite Fenton reaction mechanism generating no OH radicals, which is consistent with conventional experimental findings as far as we know, is successfully revealed.

The approach of H$_2$O$_2$ coordinating to the Fe cation hydration complexes to a Nafion side chain has first been discussed as the initial process of the Fenton reaction. For the Fe cation hydration complex, monovalent Fe cation (Fe$^+$) complex has been examined besides ferrous ion one (Fe$^{2+}$), because Fe$^+$ complex is reported to drive H$_2$O$_2$ decomposition^27^. As a result, it has been found that the Fenton reaction requires the activation energy of about 30 kcal/mol to proceed for the ferrous ion complex, while it significantly depends on the hydration number and needs the O–O bond extension of H$_2$O$_2$ for the Fe$^+$ complex. This activation energy is close to the experimental value^51^. Note that the O–O bond extension spontaneously takes place in H$_2$O$_2$-coordinated Fe$^+$ hydration complex^14^. The result also shows that the Fenton reaction does not go through OH radical formation from H$_2$O$_2$, though this reaction has been explained to generate OH radicals.

Next, the C–S bond dissociations of a Nafion side chain has been explored for both ferrous and Fe$^+$ ion hydration complexes. The calculated results suggest that the C–S bond dissociation spontaneously proceeds after the H$_2$O$_2$ approach and accompanies the C–F bond dissociation for both the ferrous and Fe$^+$ ion hydration complexes in high humidity. Different products are given for these complexes: carbonic acid –C(=O)OH group and sulfonic acid HSO$_4$ for ferrous ion and trihydroxymethyl –C(OH)$_3$ group and sulfur trioxide SO$_3$ for Fe$^+$. Note, however, that the products of Fe$^+$ hydration complex are rapidly transformed into the same products of ferrous ion hydration complex in aqueous solution.

The decomposition products in the Fenton reaction have been confirmed by comparing the vibrational spectra of the products to the experimental IR spectrum after the Fenton test. It has consequently been found that the IR spectrum is well reproduced by the vibrational spectrum of the product of the decomposition by the ferrous ion hydration complex. In particular, it has been found that two newly-appeared peaks after the decomposition correspond to the C–O asymmetric stretching of the –C(=O)OH group and the S–O stretching of the SO$_4$H fragment.

Finally, the schematic diagram of Nafion decomposition and related reactions by H$_2$O$_2$ has been summarized. As a result, it is found that the diagram is consistent with all experimental findings, as far as we know. The schematic diagram of the collective reaction mechanisms related to the decomposition of Nafion membrane is also drawn. Consequently, it is suggested that the recycle of Fe ion hydration complexes also accelerate the Fenton reaction. In conclusion, the Fenton reaction mechanism of Nafion membrane is comprehensively revealed.

## Supplementary information

Supplementary Information. The Cartesian coordinates of the hydrated Nafion decomposition models resulting from the bond dissociations for the hydration numbers of λ=3,4 and 5 have been provided.
