# Supplementary material for: Fenton reaction mechanism generating no OH radicals in Nafion membrane decomposition
Source: Sci Rep. 2020 Oct 23;10:18144. doi: 10.1038/s41598-020-74646-0 (PMC7584670; doi:10.1038/s41598-020-74646-0)
Supplement: Supplementary file 1 — Supplementary Information. The Cartesian coordinates of the hydrated Nafion decomposition models resulting from the bond dissociations for the hydration numbers of λ=3,4 and 5 have been provided. [file 41598_2020_74646_MOESM1_ESM.pdf]

Supporting Information

Fenton reaction mechanism generating no OH radicals in Nafion membrane decomposition

Takao Tsuneda[1,2*]

[1]1Graduate School of Science, Technology, and Innovation, Kobe University, Kobe 657-8501, Japan

[2]Department of Chemistry, Faculty of Science, Hokkaido University, Sapporo 060-0810, Japan

* Corresponding author: tsuneda@phoenix.kobe-u.ac.jp

Fig. S1. Cartesian coordinates of the optimized geometries of doubly-hydrated Nafion membrane model plus divalent Fe cation (Fe^{2+}) or monovalent Fe cation (Fe^{+}) hydration complexes coordinating H_2O_2. The bond lengths of dissociating C-S, approaching O-C and dissociating O-O bonds, which are shown in Fig. 1, are also summarized in tables after the coordinate tables. The unit is Å.

(λ = 3)

	Fe^{2+}				Fe^{+}		
Atom	X	Y	Z	Atom	X	Y	Z
C	-7.67473	-2.40018	-2.17348	C	-8.34261	-1.83369	-1.33284
C	-6.59987	-2.30368	-1.09823	C	-7.12155	-1.80365	-0.42361
C	-5.20278	-1.97477	-1.64032	C	-5.79078	-1.6909	-1.18617
C	-4.05228	-2.12557	-0.61838	C	-4.53804	-2.02346	-0.34998
C	-2.67697	-1.83356	-1.25931	C	-3.27513	-2.11419	-1.24667
C	-1.49469	-1.87875	-0.25659	C	-1.93828	-2.05563	-0.48637
F	-2.68308	-0.59309	-1.79579	F	-3.28764	-1.12214	-2.14229
F	-2.45697	-2.71161	-2.23145	F	-3.33171	-3.28314	-1.89011
F	-1.44166	-0.67862	0.354245	F	-1.93403	-1.02141	0.347424
F	-1.67104	-2.81924	0.661553	F	-1.80325	-3.19201	0.208281
F	-4.01218	-3.38439	-0.1688	F	-4.67507	-3.2171	0.244857
F	-4.95361	-2.78346	-2.67372	F	-5.83407	-2.5134	-2.24123
F	-5.20198	-0.70579	-2.06316	F	-5.68004	-0.42854	-1.61142
F	-6.95504	-1.33082	-0.24274	F	-7.23965	-0.73991	0.383384
F	-6.56718	-3.468	-0.44446	F	-7.14914	-2.91161	0.325791
F	-7.48945	-3.51358	-2.88506	F	-8.42882	-3.02681	-1.92963
F	-7.5808	-1.3386	-2.98576	F	-8.227	-0.88406	-2.27055
O	-4.22384	-1.18103	0.38566	O	-4.42513	-0.98484	0.557802
C	-4.5635	-1.48922	1.674388	C	-4.34237	-1.17095	1.899399
C	-5.14239	-0.21233	2.327617	C	-3.95579	0.199911	2.546076
C	-4.93727	-0.19596	3.846704	C	-2.76475	0.087006	3.505485
O	-4.492	0.910614	1.838063	O	-3.56363	1.127473	1.60987
C	-5.05154	1.69305	0.8762	C	-4.48415	1.906271	0.967019
C	-3.89194	2.449564	0.231167	C	-3.92451	2.263453	-0.4193
F	-5.46177	-2.45821	1.746199	F	-5.50212	-1.59221	2.396656
F	-3.47573	-1.88278	2.342394	F	-3.42682	-2.09844	2.205198

F	-6.45623	-0.16438	2.086531	F	-4.9999	0.634304	3.270128
F	-5.31997	-1.35388	4.356677	F	-3.01299	-0.82824	4.429374
F	-3.66705	0.017441	4.138858	F	-1.65707	-0.22772	2.856072
F	-5.66837	0.772505	4.359744	F	-2.59345	1.252279	4.10187
F	-5.65494	1.0015	-0.08555	F	-5.64942	1.27788	0.811716
F	-5.92907	2.545218	1.382956	F	-4.72032	3.013827	1.664102
F	-4.33893	3.174739	-0.78647	F	-4.94119	2.556837	-1.22185
F	-2.9957	1.56182	-0.22673	F	-3.26313	1.211435	-0.91283
S	-2.97505	3.599319	1.328591	S	-2.77445	3.694166	-0.40826
O	-2.04924	4.241611	0.36216	O	-2.18764	3.605247	-1.79331
O	-2.17247	2.636557	2.238926	O	-1.76789	3.308463	0.62386
O	-3.89154	4.396024	2.090582	O	-3.5446	4.896857	-0.15666
C	-0.16056	-2.16151	-0.97441	C	-0.72514	-1.89478	-1.42112
C	1.100963	-1.9726	-0.10935	C	0.611268	-2.1785	-0.69731
C	2.388505	-2.03958	-0.96265	C	1.807852	-1.55602	-1.44967
C	3.69831	-2.3309	-0.19177	C	3.214838	-2.12897	-1.14411
C	4.862125	-2.52958	-1.20265	C	4.240656	-1.60325	-2.18038
C	6.258089	-2.31827	-0.62303	C	5.704707	-1.72503	-1.74755
C	7.356525	-2.83485	-1.54505	C	6.659236	-1.579	-2.92799
F	4.729551	-1.67053	-2.22631	F	4.020181	-0.29696	-2.44187
F	4.764854	-3.77066	-1.68097	F	4.072039	-2.28307	-3.31594
F	8.519617	-2.39939	-1.05372	F	7.89104	-1.42406	-2.43126
F	7.345485	-4.17103	-1.52069	F	6.627131	-2.69984	-3.65692
F	6.441549	-1.0047	-0.44559	F	5.978936	-0.75562	-0.87055
F	6.3317	-2.94593	0.555914	F	5.893802	-2.91546	-1.16887
F	3.530083	-3.47869	0.473288	F	3.185802	-3.46085	-1.26275
F	2.251338	-2.98844	-1.88864	F	1.601606	-1.70839	-2.76442
F	2.478954	-0.8475	-1.57846	F	1.825967	-0.24393	-1.16193
F	1.079591	-0.74647	0.454183	F	0.575511	-1.66376	0.531368
F	1.085369	-2.88734	0.849134	F	0.763516	-3.50101	-0.61499
F	-0.1849	-3.42572	-1.37964	F	-0.83313	-2.75102	-2.44117
F	-0.07705	-1.33735	-2.03435	F	-0.71263	-0.64762	-1.89788
O	3.996802	-1.26328	0.64411	O	3.647112	-1.70313	0.1012
C	4.000865	-1.32894	2.012449	C	3.739008	-2.51697	1.196483
C	4.694854	-0.03326	2.548915	C	4.427844	-1.69471	2.334798

C	6.123183	-0.28472	3.059504	C	5.88644	-2.12284	2.550532
O	4.8282	0.94014	1.575167	O	4.503704	-0.34808	2.070299
C	3.760503	1.66311	1.174649	C	3.392424	0.438105	2.143699
C	3.704045	1.766429	-0.36929	C	3.44553	1.476827	0.998462
F	2.74984	-1.40178	2.474111	F	2.541845	-2.93894	1.586891
F	4.643809	-2.39662	2.464447	F	4.463032	-3.60531	0.93425
F	3.985323	0.412511	3.596598	F	3.765328	-1.9402	3.476705
F	6.076525	-1.05675	4.128651	F	5.931617	-3.361	3.010999
F	6.861634	-0.85681	2.12816	F	6.56326	-2.04705	1.416114
F	6.658274	0.873272	3.39077	F	6.443779	-1.32432	3.439784
F	2.609146	1.092339	1.590296	F	2.283574	-0.29613	2.0037
F	3.790732	2.884846	1.70396	F	3.344089	1.041209	3.325178
F	4.421792	0.807566	-0.93423	F	4.207762	0.985619	0.009521
F	2.404199	1.593324	-0.71323	F	2.216735	1.64642	0.49303
H	-8.66444	-2.41651	-1.68904	H	-9.24303	-1.64968	-0.72473
H	7.225171	-2.47051	-2.57582	H	6.399352	-0.71117	-3.55403
S	4.238869	3.348229	-1.15366	S	4.090415	3.162375	1.391707
O	5.587981	3.599588	-0.70788	O	5.269656	2.98512	2.212748
O	3.185057	4.331321	-0.69465	O	2.911601	3.831548	2.015299
O	4.008796	3.032876	-2.5888	O	4.314335	3.676085	0.000167
H	0.159409	0.753794	1.487672	H	0.258975	0.972501	1.204931
O	1.793524	1.786567	-3.23306	O	3.010366	2.487145	-2.02218
H	2.701966	2.178496	-3.08043	H	3.720995	2.748381	-1.38925
H	1.804394	0.935233	-2.76518	H	2.879734	1.531642	-1.91268
O	-1.49714	4.316743	-2.25331	O	-0.19259	5.18532	-2.23094
H	-1.9681	4.351206	-1.39108	H	-1.07601	4.724122	-2.1188
H	-1.49052	5.219624	-2.607	H	-0.20423	5.965936	-1.65571
O	-0.31721	2.667731	-4.39069	O	1.627359	4.183849	-3.80488
H	-1.01137	2.910133	-5.01925	H	0.818538	4.71483	-3.92366
H	0.348603	2.098161	-4.81258	H	1.583068	3.431975	-4.41363
O	1.586478	4.658905	-2.74648	O	2.802763	5.294214	-1.4482
H	2.213338	4.605123	-3.48715	H	3.261764	5.47415	-2.28218
H	2.177006	4.732019	-1.93505	H	3.480424	4.944373	-0.81719
O	-0.54566	1.468106	-1.52825	O	0.087139	2.140759	-1.84429
H	-1.32944	0.951572	-1.77479	H	-0.83882	2.446371	-1.98121

H	-0.45385	1.428737	-0.54031	H	0.036242	1.594158	-0.99565
O	1.251194	3.684861	0.777131	O	0.97778	2.167178	2.55847
H	2.140878	4.01527	0.433155	H	1.689649	2.810852	2.80716
O	0.446098	3.912323	-0.34892	O	0.807918	4.130044	0.27471
H	-0.38714	4.266537	0.044731	H	1.479192	4.126745	0.995566
Fe	0.258251	3.134057	-2.44264	Fe	1.349502	3.755422	-1.66997
O	-0.15773	1.600041	1.128407	O	-0.34033	1.014299	0.441773
H	0.606765	2.250797	1.155285	H	-0.06437	3.810877	0.610886
H	-1.35377	2.198386	1.780059	H	-1.03351	1.646504	0.719145

Bond	Bond length (Å)
Dissociating C-S bond	1.845
Approaching O-C bond	3.318
Dissociating O-O bond	1.403

(λ = 4)

	Fe^{2+}				Fe^{+}		
Atom	X	Y	Z	Atom	X	Y	Z
C	-8.52345	-2.0436	-0.79047	C	-5.59757	-1.10717	-2.84313
C	-7.20527	-1.56969	-0.19442	C	-4.87893	-1.49594	-1.56086
C	-5.96674	-2.08149	-0.94059	C	-3.72412	-2.49321	-1.74728
C	-4.65113	-1.92918	-0.13592	C	-3.31666	-3.19854	-0.43707
C	-3.41804	-2.09785	-1.0539	C	-1.98648	-4.00018	-0.56146
C	-2.04646	-2.02696	-0.3377	C	-0.74506	-3.21012	-0.11014
F	-3.39942	-1.08683	-1.95838	F	-1.83796	-4.40217	-1.83045
F	-3.53646	-3.24446	-1.71231	F	-2.08087	-5.07891	0.212217
F	-1.78796	-0.71963	-0.14563	F	-0.84364	-1.94301	-0.51871
F	-2.06661	-2.66195	0.826495	F	-0.72321	-3.25379	1.225171
F	-4.6055	-2.89139	0.792006	F	-4.27642	-4.0902	-0.15313
F	-6.12733	-3.37734	-1.21124	F	-4.10594	-3.42717	-2.62108
F	-5.85639	-1.40231	-2.08547	F	-2.68096	-1.8235	-2.25692
F	-7.19459	-0.22788	-0.21602	F	-4.3901	-0.36107	-1.04374
F	-7.14779	-1.983	1.077605	F	-5.78824	-2.00719	-0.72532

F	-8.68812	-3.34092	-0.52558	F	-6.22834	-2.14436	-3.38086
F	-8.48486	-1.86596	-2.11813	F	-4.66888	-0.65865	-3.72938
O	-4.62563	-0.65267	0.399223	O	-3.21505	-2.213	0.531072
C	-4.42333	-0.38225	1.723256	C	-3.70334	-2.38633	1.794506
C	-4.68913	1.122441	1.895652	C	-3.36169	-1.12655	2.605891
C	-4.07723	1.674458	3.190559	C	-1.87348	-0.91808	2.907457
O	-4.11985	1.763121	0.80606	O	-3.81511	-0.05741	1.857085
C	-4.77951	2.76263	0.161772	C	-4.56208	0.934416	2.420124
C	-3.80318	3.318476	-0.88005	C	-4.6472	2.067804	1.402574
F	-5.2397	-1.05776	2.513685	F	-5.02784	-2.50608	1.78929
F	-3.17088	-0.67479	2.086471	F	-3.19864	-3.48092	2.360665
F	-6.01443	1.302093	1.939588	F	-4.00266	-1.24669	3.779359
F	-4.40533	0.887942	4.203398	F	-1.37251	-1.99204	3.504571
F	-2.76103	1.723114	3.087392	F	-1.20728	-0.696	1.787875
F	-4.53967	2.887405	3.416459	F	-1.73148	0.116195	3.708723
F	-5.85752	2.310822	-0.47173	F	-5.79763	0.496932	2.680738
F	-5.14993	3.736371	0.981095	F	-4.04573	1.383587	3.555887
F	-4.18528	4.522505	-1.25872	F	-5.24609	3.097477	1.991956
F	-3.76834	2.502848	-1.9364	F	-5.39278	1.663313	0.367992
S	-2.0733	3.438303	-0.25257	S	-3.01557	2.632649	0.751197
O	-1.43902	4.472006	-1.12408	O	-3.32365	4.032191	0.350892
O	-1.51339	2.065345	-0.58704	O	-2.75503	1.725601	-0.39745
O	-2.05989	3.754421	1.16566	O	-2.04586	2.514141	1.838558
C	-0.89815	-2.61822	-1.17113	C	0.58954	-3.75108	-0.64817
C	0.483074	-2.51424	-0.4914	C	1.79116	-2.95737	-0.10762
C	1.627605	-2.74786	-1.49835	C	3.162439	-3.56314	-0.48157
C	2.938527	-3.20613	-0.81702	C	4.326887	-2.53071	-0.51397
C	4.183723	-2.99692	-1.71248	C	4.705611	-1.93905	-1.89373
C	4.867226	-1.63315	-1.55658	C	3.653811	-1.15798	-2.67502
C	5.681044	-1.24799	-2.77868	C	4.144445	-0.64262	-4.02199
F	3.802533	-3.14165	-2.99695	F	5.125209	-2.93623	-2.68616
F	5.066914	-3.94528	-1.42986	F	5.736293	-1.12752	-1.65131
F	6.200368	-0.03139	-2.53826	F	3.173356	0.181154	-4.47714
F	6.670352	-2.13227	-2.92608	F	5.261092	0.068467	-3.87841
F	3.938106	-0.6739	-1.36715	F	2.605102	-1.95835	-2.93286

F	5.651572	-1.67415	-0.4784	F	3.240007	-0.11334	-1.94995
F	2.818657	-4.52212	-0.6013	F	5.427948	-3.19705	-0.12805
F	1.263805	-3.69437	-2.36159	F	3.44949	-4.48005	0.439093
F	1.79696	-1.60145	-2.17161	F	3.092527	-4.17805	-1.66835
F	0.638797	-1.28943	0.036962	F	1.684639	-1.71388	-0.60133
F	0.525447	-3.4222	0.479375	F	1.740615	-2.8846	1.222679
F	-1.13752	-3.89615	-1.41727	F	0.716803	-5.03549	-0.30797
F	-0.85623	-1.93124	-2.3391	F	0.591627	-3.62835	-1.97684
O	3.047738	-2.5041	0.373171	O	4.080796	-1.44595	0.310005
C	3.594155	-3.07973	1.489425	C	4.544403	-1.41014	1.594066
C	3.642092	-1.97401	2.54998	C	3.816977	-0.24199	2.28325
C	4.227495	-2.42333	3.89146	C	4.318162	-0.00327	3.716243
O	4.445298	-0.93739	2.07885	O	3.986078	0.825622	1.44546
C	3.919112	0.294214	1.910608	C	3.471466	2.081229	1.701229
C	5.035588	1.226005	1.425845	C	3.539038	2.851119	0.350886
F	2.831802	-4.06896	1.941542	F	4.272655	-2.55247	2.221911
F	4.806036	-3.55971	1.243565	F	5.856817	-1.21339	1.633241
F	2.376	-1.57777	2.760365	F	2.506813	-0.59526	2.365846
F	3.492724	-3.38824	4.409967	F	4.359065	-1.14882	4.378021
F	5.464609	-2.84918	3.725945	F	5.529782	0.519135	3.685755
F	4.225688	-1.38652	4.710296	F	3.499925	0.818415	4.346106
F	2.935286	0.252933	0.976982	F	2.25646	2.011056	2.22538
F	3.393187	0.783385	3.027229	F	4.23861	2.712144	2.585006
F	6.061546	1.136975	2.251873	F	4.678017	3.548752	0.387055
F	5.406779	0.866751	0.199029	F	3.618752	2.03413	-0.69732
H	-9.34946	-1.45724	-0.3567	H	-6.3072	-0.2939	-2.62377
H	5.051176	-1.20799	-3.67648	H	4.291791	-1.46199	-4.73926
S	4.517023	3.009901	1.365383	S	2.20929	4.102219	0.020511
O	4.968668	3.633003	2.586184	O	2.890551	5.087578	-0.82096
O	5.12058	3.497096	0.079312	O	1.158286	3.319666	-0.73501
O	3.014252	2.952905	1.191443	O	1.711294	4.542412	1.332739
O	0.914896	1.041315	-2.7862	O	-0.41175	0.289556	-1.89862
H	-0.42412	0.726719	-2.85972	H	-1.31181	0.549441	-2.30234
H	1.165456	1.013155	-1.83313	H	-0.38103	0.581294	-0.91344
H	1.745043	0.739944	-3.29392	H	0.337101	0.845537	-2.35155

O	3.212834	0.447819	-3.80774	O	1.307718	1.831323	-2.8765
H	3.44595	0.787199	-4.68548	H	2.158912	1.467664	-3.16755
H	3.827607	0.896168	-3.17944	H	1.470081	2.417687	-2.08206
O	3.040759	4.843432	-3.51462	O	-0.95693	3.335299	-3.23861
H	3.33218	5.48988	-2.8475	H	-1.62719	2.627305	-3.29516
H	2.069195	4.833885	-3.4213	H	-0.10912	2.936512	-3.50622
O	1.017905	1.70325	-0.19204	O	-0.80789	5.241649	0.551131
H	0.023566	1.739022	-0.11132	H	-1.62196	4.896736	0.961511
H	1.40884	1.215426	0.557334	H	-0.03672	4.990796	1.106926
O	0.721842	4.860382	0.522193	O	-0.97611	7.538633	-1.02631
H	0.490428	4.734621	1.457684	H	-1.74619	7.103003	-1.43641
H	-0.11933	5.051552	0.048748	H	-0.85894	6.995189	-0.22341
O	3.323686	5.258657	-0.72787	O	0.727796	5.95114	-2.35119
H	3.171932	6.008638	-0.12906	H	0.297263	6.753158	-1.96741
H	4.191353	4.839129	-0.46786	H	1.612501	5.878484	-1.94719
O	0.811955	3.774911	-2.26985	O	-2.73505	5.451822	-1.78879
H	-0.12096	4.036682	-2.05026	H	-3.18048	4.948837	-1.06404
H	0.793222	2.879366	-2.68389	H	-3.13834	5.184605	-2.6256
O	3.217555	2.804406	-2.07224	O	1.563659	0.887903	0.06164
H	3.280049	3.566299	-2.76351	H	1.72659	0.120385	0.636171
O	4.441065	2.131158	-2.12068	O	0.211312	1.14844	0.375075
H	4.92846	2.483254	-1.33067	H	0.202253	2.134988	0.29363
Fe	2.016617	3.564177	-0.494	Fe	-0.72002	4.551872	-1.52367
O	-1.49546	0.756279	-2.73219	O	-2.60791	1.169694	-2.83719
H	-1.91399	-0.12228	-2.65843	H	-3.2663	0.570916	-3.22302
H	-1.67917	1.29333	-1.87728	H	-2.92135	1.444055	-1.92451

Bond	Bond length (Å)
Dissociating C-S bond	1.859
Approaching O-C bond	3.708
Dissociating O-O bond	1.397

(λ = 5)

	Fe^{2+}				Fe^{+}		
Atom	X	Y	Z	Atom	X	Y	Z
C	-9.39509	-0.88397	-0.77414	C	-8.68456	-0.74157	-1.82275
C	-8.05126	-0.67692	-0.0883	C	-7.56137	-0.76703	-0.79498
C	-6.85273	-1.17672	-0.90682	C	-6.15936	-0.8493	-1.43101
C	-5.52176	-1.23479	-0.12905	C	-5.02878	-1.48159	-0.58593
C	-4.40514	-1.8935	-0.97807	C	-3.77131	-1.75121	-1.45552
C	-2.98556	-1.71091	-0.41462	C	-2.49303	-1.89037	-0.60196
F	-4.39112	-1.39925	-2.21966	F	-3.55216	-0.73643	-2.3172
F	-4.67315	-3.19877	-1.02282	F	-3.97426	-2.86105	-2.15832
F	-2.66375	-0.40812	-0.45342	F	-2.09068	-0.65919	-0.27553
F	-2.94288	-2.15015	0.841273	F	-2.76011	-2.58472	0.504918
F	-5.65159	-1.99756	0.96506	F	-5.42486	-2.67348	-0.12306
F	-7.12685	-2.39987	-1.37066	F	-6.2487	-1.59607	-2.54452
F	-6.6827	-0.34042	-1.934	F	-5.79998	0.389685	-1.76559
F	-7.89071	0.64006	0.121649	F	-7.64108	0.371871	-0.09498
F	-8.09675	-1.30139	1.092563	F	-7.77864	-1.79869	0.027249
F	-9.70415	-2.18307	-0.75775	F	-8.84248	-1.95636	-2.35518
F	-9.31403	-0.46833	-2.04483	F	-8.3681	0.128764	-2.7921
O	-5.17432	0.074879	0.175049	O	-4.61239	-0.63431	0.422683
C	-5.05958	0.495649	1.46621	C	-4.99745	-0.79835	1.72097
C	-4.84363	2.014452	1.421222	C	-4.24499	0.243491	2.612258
C	-4.39718	2.575801	2.780481	C	-3.10509	-0.41463	3.398839
O	-3.85204	2.247961	0.479427	O	-3.62931	1.256189	1.914302
C	-3.92727	3.299152	-0.3786	C	-4.36355	2.295231	1.423056
C	-2.5724	3.357825	-1.08467	C	-3.60216	2.918302	0.25737
F	-6.15349	0.235382	2.172254	F	-6.30934	-0.65598	1.862827
F	-4.02611	-0.09163	2.073165	F	-4.69624	-2.02924	2.137967
F	-6.00153	2.589911	1.074977	F	-5.12588	0.734654	3.49675
F	-5.18826	2.114747	3.736796	F	-3.59269	-1.288	4.26132
F	-3.15426	2.212442	3.032301	F	-2.27452	-1.02959	2.567716
F	-4.47081	3.89243	2.763185	F	-2.44274	0.511239	4.061873

F	-4.87462	3.119582	-1.29449	F	-5.5619	1.88782	0.984367
F	-4.15722	4.452776	0.234071	F	-4.57587	3.208768	2.355724
F	-2.43373	4.530428	-1.69337	F	-4.29929	3.962059	-0.17018
F	-2.49831	2.379075	-1.98341	F	-3.49446	2.027499	-0.73926
S	-1.16103	3.159524	0.07858	S	-1.89851	3.480642	0.678356
O	-0.00557	3.7156	-0.71823	O	-1.7047	4.586656	-0.30509
O	-0.94762	1.672523	0.172162	O	-1.0483	2.278549	0.360608
O	-1.47836	3.841629	1.312093	O	-1.88306	3.88236	2.072907
C	-1.90023	-2.45303	-1.22563	C	-1.3665	-2.63778	-1.33518
C	-0.56017	-2.55551	-0.47051	C	-0.00414	-2.56393	-0.62478
C	0.608659	-2.91688	-1.41537	C	1.131344	-3.15496	-1.4915
C	1.813687	-3.52419	-0.66588	C	2.285031	-3.66941	-0.61518
C	3.102004	-3.62443	-1.52468	C	3.607321	-3.99725	-1.35763
C	4.054969	-2.42324	-1.46921	C	4.673608	-2.89736	-1.39635
C	4.98914	-2.41527	-2.67082	C	5.668223	-3.10434	-2.52634
F	2.732819	-3.79179	-2.81156	F	3.302446	-4.35881	-2.61996
F	3.770502	-4.70068	-1.13238	F	4.149867	-5.0452	-0.74593
F	5.884186	-1.41781	-2.50336	F	6.617493	-2.16745	-2.40193
F	5.649991	-3.57427	-2.71608	F	6.232806	-4.31041	-2.3757
F	3.333462	-1.28071	-1.50785	F	4.100316	-1.68728	-1.55638
F	4.739525	-2.45704	-0.3296	F	5.307486	-2.93047	-0.22719
F	1.466105	-4.77971	-0.35292	F	1.854052	-4.82928	-0.09664
F	0.183889	-3.80125	-2.31351	F	0.656586	-4.1736	-2.20687
F	0.965639	-1.78982	-2.06096	F	1.553844	-2.203	-2.33294
F	-0.29621	-1.3656	0.0887	F	0.304833	-1.29625	-0.33936
F	-0.67714	-3.48055	0.475448	F	-0.11749	-3.26075	0.507023
F	-2.29257	-3.66458	-1.57452	F	-1.72725	-3.91455	-1.42168
F	-1.67368	-1.72626	-2.35563	F	-1.22542	-2.12852	-2.57513
O	2.013293	-2.74499	0.461103	O	2.478272	-2.69315	0.348089
C	2.377013	-3.318	1.653825	C	2.779096	-3.03381	1.630217
C	2.596049	-2.15417	2.627639	C	3.035017	-1.74599	2.435199
C	2.689415	-2.60898	4.089898	C	3.376303	-2.07537	3.889717
O	3.793069	-1.54343	2.282134	O	4.121501	-1.04126	1.933073
C	3.849109	-0.19933	2.11161	C	4.078456	0.296234	1.648576
C	5.350549	0.173061	2.15387	C	5.514413	0.681295	1.349279

F	1.409049	-4.09753	2.125708	F	1.756995	-3.67881	2.186115
F	3.476044	-4.0465	1.516952	F	3.83828	-3.83408	1.667276
F	1.543617	-1.32839	2.509818	F	1.901893	-1.02173	2.430353
F	1.500406	-2.96961	4.531265	F	4.46659	-2.81767	3.946637
F	3.523587	-3.63043	4.183922	F	3.581984	-0.94682	4.543192
F	3.140359	-1.61013	4.825813	F	2.375946	-2.72773	4.4592
F	3.182249	0.139523	0.995706	F	3.242325	0.609225	0.635267
F	3.269168	0.473261	3.10703	F	3.666646	0.979183	2.71614
F	5.691919	-0.04274	3.425906	F	6.129596	0.540771	2.539568
F	6.114823	-0.61358	1.412969	F	6.026214	-0.26355	0.526487
H	-10.1632	-0.30357	-0.23778	H	-9.61442	-0.42356	-1.32446
H	4.423955	-2.23495	-3.59379	H	5.175392	-3.01958	-3.50143
S	5.72465	1.998011	1.899065	S	5.921709	2.426384	0.659068
O	6.179941	2.497127	3.173379	O	6.996403	2.763094	1.417067
O	6.729731	2.031177	0.773313	O	6.243906	2.201809	-0.79149
O	4.424758	2.59609	1.415702	O	4.728419	3.335814	0.893412
O	2.093403	0.951237	-2.64838	O	1.427532	1.286836	-3.37095
H	0.679751	0.752732	-2.23702	H	0.576888	1.123015	-2.79711
H	2.654533	0.765391	-1.86729	H	2.014693	1.997866	-2.9448
H	2.468648	0.376645	-3.38738	H	2.069691	0.524877	-3.65297
O	3.387022	-0.38509	-4.45513	O	3.366231	-0.14281	-4.22238
H	3.161481	-0.47027	-5.39284	H	3.408423	-0.0778	-5.18795
H	4.234733	0.123368	-4.39407	H	4.149284	0.412169	-3.88962
O	5.603812	3.500478	-3.617	O	1.880257	5.959093	-2.86373
H	6.010726	3.739055	-2.76143	H	1.063932	5.484425	-3.10563
H	4.654855	3.683921	-3.46236	H	2.60926	5.423968	-3.20953
O	2.068943	2.217942	-0.17073	O	1.415078	3.391597	0.968426
H	1.260339	2.778012	-0.33412	H	0.765211	2.662799	1.003278
H	1.965613	1.728777	0.662703	H	2.275583	3.084989	1.297196
O	3.420037	5.031217	0.074235	O	2.478598	7.533422	-0.64304
H	3.281883	5.186688	1.021491	H	2.23443	7.222502	-1.5347
H	2.684456	5.44813	-0.44318	H	1.722156	7.2337	-0.10425
O	5.89102	3.855959	-0.85847	O	3.50732	5.165767	-0.35977
H	5.997804	4.756865	-0.50878	H	3.359437	6.153545	-0.35299
H	6.431323	3.244113	-0.26471	H	3.777729	4.885737	0.530153

O	3.071943	3.493351	-2.47382	O	-0.11258	4.170975	-2.3821
H	2.429988	4.247221	-2.41127	H	-0.82138	4.447892	-1.74496
H	2.578334	2.669661	-2.72161	H	-0.3311	3.235238	-2.55573
O	4.599465	1.248135	-1.312	O	4.034152	2.625083	-1.61457
H	4.980496	1.049123	-2.23168	H	4.482716	3.391271	-1.12292
O	5.225432	0.348085	-0.43721	O	2.832887	3.072633	-2.28207
H	6.073109	0.811004	-0.22081	H	2.371867	2.686295	-1.48581
Fe	3.912428	3.08813	-0.51756	Fe	1.575471	4.483414	-0.95196
O	-0.31848	0.722466	-1.96951	O	-0.71957	1.436077	-2.06586
H	-0.73139	-0.13793	-2.15846	H	-1.56771	1.037161	-2.32107
H	-0.59132	1.107188	-0.97957	H	-0.82334	1.703106	-1.09247
O	1.520659	5.531189	-1.68627	O	0.544864	5.925431	0.522862
H	1.231065	6.341357	-2.13214	H	0.744779	5.25143	1.204693
H	0.716411	5.057718	-1.34355	H	-0.40394	5.7835	0.324793
O	5.52247	0.979173	-3.69363	O	5.301411	1.381794	-3.31727
H	6.364827	0.497583	-3.71582	H	5.155302	1.515131	-2.31936
H	5.684258	1.982698	-3.8103	H	6.218645	1.102799	-3.44054

Bond	Bond length (Å)
Dissociating C-S bond	1.880
Approaching O-C bond	2.600
Dissociating O-O bond	1.403

Fig. S2. Cartesian coordinates of the optimized geometries of doubly-hydrated Nafion membrane model plus divalent Fe cation (Fe^{2+}) hydration complex coordinating H_2O_2 for the geometrical change without and with the C-F bond dissociation of Nafion side chain with keeping the C-S distance at 6.0Å. The bond lengths of dissociating C-S, approaching O-C and dissociating O-O bonds, which are shown in Fig. 1, are also summarized in tables after the coordinate tables. The unit is Å.

(λ = 3)

	Without C-F dissociation				With C-F dissociation		
Atom	X	Y	Z	Atom	X	Y	Z
C	-8.38899	-0.50723	-1.23628	C	-9.15993	1.423105	1.166779
C	-7.00144	-0.37352	-0.62791	C	-7.90671	0.888297	0.487668
C	-5.98479	-1.3897	-1.17235	C	-6.60818	1.539999	0.975157
C	-4.80384	-1.63362	-0.20091	C	-5.39642	1.285037	0.054336
C	-3.64081	-2.3769	-0.89337	C	-4.07976	1.698081	0.76915
C	-2.41611	-2.6657	0.01093	C	-2.88553	1.933467	-0.17009
F	-3.18323	-1.63889	-1.92897	F	-3.75222	0.716329	1.615246
F	-4.12146	-3.52029	-1.37477	F	-4.27218	2.826192	1.457806
F	-1.61369	-1.58809	-0.04033	F	-2.61709	0.782068	-0.79765
F	-2.79991	-2.86117	1.267885	F	-3.18406	2.871517	-1.06651
F	-5.24617	-2.43144	0.775986	F	-5.55754	1.992747	-1.06945
F	-6.59474	-2.55972	-1.37035	F	-6.80528	2.859406	1.041225
F	-5.52592	-0.93384	-2.33958	F	-6.31408	1.068374	2.189307
F	-6.55193	0.861949	-0.89458	F	-7.84308	-0.43072	0.716787
F	-7.10596	-0.51694	0.701442	F	-8.02703	1.094843	-0.83059
F	-8.98563	-1.59928	-0.75463	F	-9.42829	2.64879	0.711297
F	-8.25968	-0.62241	-2.5657	F	-8.94548	1.481999	2.488478
O	-4.36601	-0.38474	0.220482	O	-5.31188	-0.08006	-0.17936
C	-4.17397	0.020156	1.516046	C	-5.30399	-0.6691	-1.40645
C	-4.46744	1.53253	1.599338	C	-4.76021	-2.08392	-1.16721
C	-3.92956	2.131028	2.921082	C	-4.53202	-2.89419	-2.44552
O	-3.85744	2.081671	0.491688	O	-3.523	-1.94562	-0.54135
C	-4.14718	3.322776	0.020655	C	-3.27691	-2.54596	0.64578
C	-3.11167	3.638775	-1.06537	C	-1.81058	-2.26261	0.969036

F	-4.96227	-0.60962	2.369967	F	-6.52492	-0.74296	-1.92176
F	-2.90449	-0.19506	1.871419	F	-4.52775	-0.03219	-2.28076
F	-5.79497	1.711626	1.575546	F	-5.63651	-2.74114	-0.39888
F	-4.05601	1.249082	3.902809	F	-5.63884	-2.93112	-3.16659
F	-2.65523	2.44344	2.788642	F	-3.56229	-2.35452	-3.16031
F	-4.6172	3.20907	3.244633	F	-4.19093	-4.12406	-2.10806
F	-5.35754	3.365963	-0.52742	F	-4.02008	-2.04733	1.628411
F	-4.08796	4.253374	0.966896	F	-3.45777	-3.86309	0.612903
F	-3.18159	4.923935	-1.37678	F	-1.48645	-2.833	2.120418
F	-3.35487	2.896372	-2.14129	F	-1.62033	-0.94824	1.059434
S	-1.38689	3.274838	-0.54929	S	-0.64643	-2.93155	-0.3294
O	-0.53212	3.89223	-1.58725	O	0.397529	-3.63532	0.458167
O	-1.3942	1.743788	-0.58041	O	-0.02183	-1.62824	-0.90586
O	-1.17969	3.757919	0.804327	O	-1.36238	-3.65217	-1.34762
C	-1.62865	-3.90191	-0.46248	C	-1.61772	2.412435	0.572352
C	-0.21653	-4.04407	0.170031	C	-0.34893	2.300288	-0.29468
C	0.926229	-3.49595	-0.70606	C	0.914996	2.858751	0.38213
C	2.241605	-3.26969	0.089479	C	2.199474	2.796622	-0.47964
C	3.457885	-3.24074	-0.86693	C	3.451486	3.041518	0.401298
C	4.776404	-2.72165	-0.28996	C	4.697904	3.471227	-0.37215
C	5.900775	-2.73594	-1.3251	C	5.960674	3.41517	0.482406
F	3.15612	-2.48901	-1.9308	F	3.748721	1.889333	1.026449
F	3.702767	-4.49427	-1.27495	F	3.201093	3.977792	1.313086
F	5.783232	-1.62166	-2.09628	F	7.001791	3.466331	-0.36983
F	7.062775	-2.63644	-0.66753	F	5.982453	4.483009	1.275381
F	4.645965	-1.46184	0.144469	F	4.873587	2.604176	-1.40588
F	5.140825	-3.51432	0.719195	F	4.531332	4.688232	-0.86789
F	2.410571	-4.2739	0.961021	F	2.112345	3.736886	-1.42584
F	1.147531	-4.37159	-1.68557	F	0.704784	4.127372	0.709945
F	0.551391	-2.32822	-1.24783	F	1.138779	2.134624	1.495695
F	-0.21141	-3.41682	1.353171	F	-0.15106	0.990461	-0.51319
F	0.015391	-5.33677	0.368801	F	-0.52291	2.924053	-1.45686
F	-2.32883	-4.98078	-0.137	F	-1.7983	3.672369	0.949999
F	-1.50607	-3.84285	-1.79887	F	-1.40559	1.64877	1.65775
O	2.105389	-2.04484	0.709708	O	2.362204	1.52031	-1.0001

C	2.404255	-1.78228	2.011705	C	2.340262	1.18063	-2.31956
C	2.480366	-0.23067	2.143759	C	3.030665	-0.19966	-2.37308
C	3.704293	0.246172	2.932893	C	2.678693	-1.00088	-3.63026
O	2.6027	0.374987	0.916099	O	4.366808	0.084524	-2.251
C	1.494055	0.800798	0.237663	C	5.361476	-0.83126	-2.04325
C	1.928557	1.041661	-1.21816	C	6.421223	-0.18504	-1.14153
F	1.482688	-2.25188	2.836631	F	1.096002	1.095568	-2.78047
F	3.569136	-2.33226	2.371849	F	2.99867	2.030109	-3.09248
F	1.397975	0.192554	2.812404	F	2.589792	-0.92966	-1.30972
F	3.721569	-0.27769	4.137001	F	1.414837	-1.36536	-3.59559
F	4.833739	-0.01732	2.304696	F	2.912203	-0.27171	-4.70216
F	3.612804	1.581812	3.052521	F	3.432685	-2.09492	-3.67069
F	0.479156	-0.05609	0.327651	F	4.899349	-1.94325	-1.43248
F	1.079589	1.961012	0.786245	F	5.911191	-1.19972	-3.18515
F	1.923837	-0.19322	-1.81989	F	5.708667	0.248696	-0.01301
F	0.885083	1.695011	-1.84896	F	3.457739	-0.84498	2.204085
H	-8.98195	0.389202	-0.99365	H	-10.0026	0.745619	0.954102
H	5.897579	-3.62626	-1.96789	H	6.019143	2.490552	1.071802
S	7.848261	0.916566	-0.24787	S	3.115709	-0.1522	3.865716
O	9.169498	1.357124	0.084639	O	2.014461	-1.05075	4.204222
O	7.295474	-0.13954	0.811255	O	2.810749	1.205432	3.508837
O	6.732599	1.859743	-0.38854	O	4.409434	-0.46865	4.462232
H	-1.6314	-0.65925	-2.11359	H	0.594757	0.053247	1.542347
O	5.206566	3.452514	-2.04662	O	4.468093	-3.07162	3.97987
H	5.842979	2.798368	-1.69264	H	4.659959	-2.16584	4.334182
H	5.661241	4.309229	-2.03265	H	3.940966	-3.52172	4.658166
O	1.689978	4.46632	0.083232	O	3.020923	-3.35001	0.042734
H	0.897418	4.555446	-0.48903	H	2.05163	-3.54359	0.022238
H	1.31917	4.099699	0.904178	H	3.19952	-2.71247	-0.66634
O	3.813094	5.654897	-1.22852	O	4.447746	-5.00207	1.702831
H	3.231979	5.880165	-1.97782	H	4.190794	-5.48699	0.902925
H	3.495832	6.185079	-0.47863	H	4.951474	-5.58589	2.288397
O	4.528671	3.206257	0.753357	O	5.537386	-2.16856	1.338242
H	4.145601	2.776494	1.53576	H	5.446255	-1.19852	1.300665
H	5.344435	2.704085	0.542877	H	6.166972	-2.3948	0.63237

O	2.057848	4.075886	-2.68112	O	1.86676	-3.21355	2.696545
H	1.116698	3.859112	-2.49024	H	1.126897	-3.32715	2.058884
H	2.298852	3.520242	-3.43944	H	1.650255	-2.45911	3.296595
O	7.944869	0.058585	-1.56972	O	7.063595	0.851277	-1.68652
H	7.085505	-0.36866	-1.78432	H	6.474671	1.624496	-1.77412
O	3.029581	1.67447	-1.33114	O	7.285051	-1.15148	-0.76759
H	8.021868	-0.72246	1.106369	H	8.168822	-0.75372	-0.67247
Fe	3.346126	3.555221	-0.97228	Fe	3.77935	-3.06184	2.001599
O	-1.04206	0.08939	-2.30651	O	1.048521	-0.71657	1.161888
H	-0.12593	-0.23997	-2.28641	H	2.004785	-0.5749	1.312005
H	-1.21812	1.098058	-1.44999	H	0.489156	-1.14742	-0.13709

Bond	Bond length (Å)
Dissociating C-S bond	6.000
Approaching O-C bond	1.275
Dissociating O-O bond	5.180

(λ = 4)

	Without C-F dissociation				With C-F dissociation		
Atom	X	Y	Z	Atom	X	Y	Z
C	-8.59154	0.910993	-2.16322	C	-8.31112	0.262697	-1.63336
C	-7.46818	0.417474	-1.26115	C	-7.12669	0.034114	-0.70339
C	-6.17303	0.077144	-2.01044	C	-5.79976	-0.19281	-1.45832
C	-5.11716	-0.68156	-1.18086	C	-4.69866	-1.0166	-0.74384
C	-3.93678	-1.14318	-2.07937	C	-3.55824	-1.38633	-1.72349
C	-2.68972	-1.54637	-1.2813	C	-2.30444	-1.94305	-1.00295
F	-3.56532	-0.17201	-2.91894	F	-3.16975	-0.29253	-2.4017
F	-4.35813	-2.19441	-2.78031	F	-3.99736	-2.29784	-2.58584
F	-2.19804	-0.43129	-0.71074	F	-1.56669	-0.90285	-0.58182
F	-3.02686	-2.42901	-0.34366	F	-2.67105	-2.69116	0.032119
F	-5.64139	-1.79802	-0.65886	F	-5.21791	-2.16421	-0.29335
F	-6.47563	-0.66533	-3.0805	F	-6.07655	-0.8469	-2.59599
F	-5.62087	1.226246	-2.40933	F	-5.29658	1.004378	-1.75791

F	-7.19038	1.390316	-0.37676	F	-6.99764	1.134011	0.051272
F	-7.91752	-0.65399	-0.60121	F	-7.41329	-1.00442	0.087375
F	-9.06683	-0.11138	-2.87846	F	-8.66124	-0.88771	-2.2123
F	-8.11467	1.845187	-2.99644	F	-7.95291	1.14452	-2.57725
O	-4.65325	0.194167	-0.20369	O	-4.0741	-0.27207	0.247093
C	-4.84881	-0.04762	1.123566	C	-4.35837	-0.38493	1.577066
C	-4.45933	1.223332	1.892291	C	-3.40328	0.589536	2.32829
C	-4.41282	0.967938	3.423431	C	-2.20582	-0.13852	2.956266
O	-3.21131	1.598336	1.406404	O	-2.84122	1.527862	1.489339
C	-2.78299	2.875201	1.563963	C	-3.549	2.627761	1.110353
C	-1.30647	2.93698	1.179116	C	-2.92734	3.150959	-0.18516
F	-6.11936	-0.33139	1.385453	F	-5.62051	-0.08061	1.841324
F	-4.09395	-1.06126	1.549948	F	-4.1563	-1.62981	2.013251
F	-5.38058	2.16135	1.642469	F	-4.09451	1.168974	3.318574
F	-5.25514	0.002954	3.757629	F	-2.60163	-0.87337	3.974537
F	-3.19322	0.6117	3.778354	F	-1.60658	-0.91135	2.060013
F	-4.76015	2.061707	4.073525	F	-1.33982	0.763447	3.391671
F	-3.43765	3.717692	0.76705	F	-4.82698	2.328383	0.871348
F	-2.91473	3.308255	2.813478	F	-3.51406	3.572609	2.038388
F	-0.85578	4.162396	1.486381	F	-3.40701	4.355036	-0.44386
F	-1.18023	2.767157	-0.14288	F	-3.22478	2.316743	-1.18112
S	-0.17854	1.74366	2.013601	S	-1.09404	3.249465	-0.16105
O	1.143843	2.29196	1.636969	O	-0.75275	4.202359	-1.22815
O	-0.43352	0.438248	1.246134	O	-0.65347	1.844865	-0.4307
O	-0.56102	1.645828	3.397688	O	-0.68705	3.682414	1.206224
C	-1.54704	-2.15423	-2.12157	C	-1.4135	-2.78095	-1.93573
C	-0.45017	-2.75747	-1.21552	C	-0.04947	-3.21279	-1.30362
C	0.905745	-2.93141	-1.92852	C	1.145725	-2.37471	-1.77377
C	1.919213	-3.73805	-1.08974	C	2.515164	-2.7251	-1.13732
C	3.360215	-3.72221	-1.67324	C	3.64141	-2.01853	-1.96119
C	4.306673	-2.67592	-1.07721	C	4.973602	-1.77749	-1.25276
C	5.662775	-2.61832	-1.76715	C	6.122347	-1.54997	-2.24688
F	3.289522	-3.54719	-2.99871	F	3.214731	-0.84095	-2.44036
F	3.917381	-4.91286	-1.43271	F	3.903929	-2.81601	-3.00587
F	5.512938	-2.26077	-3.06129	F	5.701166	-0.92421	-3.3496

F	6.370305	-1.66072	-1.15749	F	7.035948	-0.76198	-1.63199
F	3.738652	-1.46566	-1.12122	F	4.869644	-0.72011	-0.4365
F	4.553434	-2.99866	0.1999	F	5.283108	-2.85533	-0.52496
F	1.515855	-5.0122	-1.06446	F	2.737237	-4.03598	-1.29829
F	0.685642	-3.55502	-3.08353	F	1.276457	-2.54213	-3.09488
F	1.420742	-1.7158	-2.16887	F	0.858972	-1.08696	-1.5174
F	-0.29088	-1.94485	-0.16389	F	-0.1483	-3.10523	0.025267
F	-0.86717	-3.94885	-0.7938	F	0.172469	-4.48015	-1.62638
F	-1.99127	-3.08812	-2.94522	F	-2.04753	-3.87745	-2.3066
F	-1.00728	-1.149	-2.85137	F	-1.18685	-2.01342	-3.03507
O	1.916028	-3.15393	0.165023	O	2.518798	-2.34487	0.198468
C	1.836116	-3.87334	1.320543	C	2.596712	-3.27646	1.211219
C	1.822042	-2.82572	2.44154	C	1.988849	-2.69902	2.503704
C	1.805542	-3.42829	3.847969	C	2.340546	-3.56344	3.727542
O	2.978874	-2.06543	2.323593	O	2.478709	-1.41847	2.73361
C	2.919897	-0.71807	2.212673	C	1.599023	-0.43157	3.063989
C	4.362962	-0.19493	2.290774	C	2.378322	0.769875	3.590429
F	0.71353	-4.58238	1.376426	F	1.910081	-4.38004	0.930254
F	2.864079	-4.70625	1.443644	F	3.862086	-3.62077	1.426356
F	0.713717	-2.08884	2.288269	F	0.657051	-2.70329	2.356547
F	0.735614	-4.18329	4.009582	F	2.096543	-4.83781	3.484196
F	2.89037	-4.15576	4.042785	F	3.616489	-3.41203	4.032437
F	1.781284	-2.44233	4.727531	F	1.605907	-3.16996	4.751609
F	2.39058	-0.33641	1.036191	F	0.889994	-0.06835	1.985202
F	2.192223	-0.16301	3.17767	F	0.746242	-0.79638	4.009622
F	5.042842	-0.86066	3.218704	F	3.480494	0.986335	2.857333
F	4.964403	-0.40439	1.110026	F	2.554036	0.563265	-0.30395
H	-9.38994	1.340109	-1.53632	H	-9.15297	0.663877	-1.04649
H	6.187741	-3.58273	-1.71359	H	6.598521	-2.50463	-2.50892
S	5.73401	3.676626	-2.08316	S	5.391341	2.431544	-1.32491
O	5.843554	2.437389	-1.26122	O	5.208882	1.57967	-2.50075
O	6.417829	3.671499	-3.35292	O	5.768923	3.827623	-1.51579
O	4.326925	4.182131	-2.07967	O	4.148944	2.355329	-0.42194
O	2.774247	1.003957	-1.71608	O	1.692847	1.438494	-2.65858
H	1.303875	0.528538	-1.34239	H	0.406778	1.108075	-2.82253

H	3.166112	1.248325	-0.85364	H	2.001074	0.957202	-1.85518
H	3.553097	0.680635	-2.27789	H	2.361718	1.235915	-3.42298
O	4.936073	0.478482	-2.94018	O	3.449745	1.037478	-4.41438
H	5.604602	0.92646	-2.38756	H	4.23958	1.321747	-3.90301
H	5.309269	-0.35618	-3.25551	H	3.637693	0.125573	-4.68018
O	2.020319	5.948166	-1.60076	O	4.236563	5.403038	-0.11095
H	1.55119	5.463957	-2.3022	H	3.927098	6.229823	-0.50874
H	2.653296	6.52706	-2.05434	H	4.918045	5.0106	-0.7167
O	3.808023	2.586187	0.324011	O	1.689483	2.416144	1.346758
H	3.776415	2.215591	1.227991	H	0.767197	2.71018	1.527188
H	4.705455	2.414266	-0.08132	H	1.619503	1.589121	0.826893
O	1.879152	4.763838	1.221565	O	1.437773	5.313464	1.063374
H	1.120722	5.36404	1.163042	H	1.593371	6.022076	1.703041
H	1.542804	3.909051	1.592986	H	0.537045	4.941075	1.221916
O	4.369349	5.732585	0.242401	O	3.903089	3.790955	2.22119
H	4.407714	5.981578	1.176922	H	3.885448	2.916793	2.649416
H	5.279336	5.644651	-0.11995	H	4.803143	4.145198	2.282366
O	1.558816	3.265682	-1.57951	O	1.893578	3.876613	-1.41581
H	0.769968	2.885497	-1.15122	H	0.969755	4.228076	-1.45496
H	2.047149	2.450728	-1.90876	H	1.883714	3.062015	-1.97337
O	6.47958	4.826669	-1.16218	O	6.538942	1.75517	-0.46029
H	7.313735	5.069657	-1.60384	H	6.699408	0.851857	-0.80665
O	4.34817	1.128317	2.588101	O	2.083576	1.440563	4.499716
H	5.160051	1.347085	3.078505	H	3.335254	1.194089	-0.33204
Fe	2.974975	4.353015	-0.49393	Fe	2.862908	3.908493	0.39661
O	0.351083	0.672881	-1.05552	O	-0.67687	0.950211	-2.74328
H	-0.2295	0.260464	-1.71389	H	-0.94535	0.020853	-2.84317
H	-0.04557	0.473607	0.237798	H	-0.86063	1.264848	-1.7778

Bond	Bond length (Å)
Dissociating C-S bond	6.000
Approaching O-C bond	1.356
Dissociating O-O bond	5.682

(λ = 5)

	Without C-F dissociation				With C-F dissociation		
Atom	X	Y	Z	Atom	X	Y	Z
C	8.772446	-1.73296	-1.95991	C	-9.22325	0.43662	-2.02598
C	7.744709	-1.22472	-0.95712	C	-8.02653	0.229861	-1.10727
C	6.581997	-0.45844	-1.60323	C	-6.76258	-0.25359	-1.83351
C	5.707418	0.393171	-0.65584	C	-5.64244	-0.75283	-0.89865
C	4.765352	1.311265	-1.47543	C	-4.44498	-1.32485	-1.69738
C	3.580092	1.849681	-0.64955	C	-3.18638	-1.58516	-0.85379
F	4.263959	0.650795	-2.51981	F	-4.08969	-0.49247	-2.68021
F	5.485553	2.345145	-1.915	F	-4.84322	-2.48596	-2.21902
F	2.630325	0.903457	-0.61995	F	-2.73926	-0.42651	-0.34925
F	3.984306	2.122344	0.591525	F	-3.49286	-2.41939	0.139882
F	6.459422	1.189977	0.111844	F	-6.09543	-1.75061	-0.12722
F	7.082798	0.358263	-2.53858	F	-7.09475	-1.24483	-2.66583
F	5.781751	-1.35864	-2.18188	F	-6.27969	0.773805	-2.53644
F	7.231255	-2.29125	-0.32163	F	-7.74626	1.40503	-0.52039
F	8.384249	-0.4567	-0.07091	F	-8.38364	-0.6483	-0.16453
F	9.505892	-0.70989	-2.40458	F	-9.69757	-0.75124	-2.40973
F	8.137768	-2.30111	-2.99427	F	-8.83807	1.12558	-3.10825
O	4.908725	-0.46304	0.090111	O	-5.22732	0.343593	-0.15448
C	5.115712	-0.73529	1.411048	C	-5.3024	0.356141	1.205229
C	4.397073	-2.06447	1.679687	C	-4.78256	1.730324	1.636447
C	4.164077	-2.35446	3.164093	C	-4.62121	1.882403	3.152694
O	3.146473	-2.00068	1.074928	O	-3.52573	1.881526	1.058819
C	2.797101	-2.89712	0.122916	C	-3.21701	2.995979	0.356235
C	1.354365	-2.56658	-0.24774	C	-1.73012	2.894907	0.023807
F	6.402737	-0.86063	1.710705	F	-6.54845	0.196034	1.635409
F	4.606027	0.217922	2.187817	F	-4.55174	-0.60239	1.749441
F	5.146235	-3.04792	1.167191	F	-5.6476	2.654258	1.20188
F	5.294865	-2.20308	3.834475	F	-5.75599	1.584306	3.762014
F	3.251727	-1.53832	3.65465	F	-3.66679	1.083137	3.589915
F	3.74534	-3.60007	3.288315	F	-4.29896	3.13351	3.422663
F	3.550492	-2.7947	-0.96887	F	-3.89797	3.073775	-0.78383

F	2.851314	-4.15461	0.551482	F	-3.42373	4.115075	1.042013
F	0.867354	-3.51803	-1.0389	F	-1.31126	4.064043	-0.45532
F	1.311201	-1.40131	-0.88631	F	-1.52502	1.950499	-0.89364
S	0.239409	-2.45803	1.235185	S	-0.67438	2.49235	1.491396
O	-1.04497	-3.01207	0.711488	O	0.607	3.15074	1.146457
O	0.09078	-0.94241	1.431707	O	-0.50944	0.959826	1.353774
O	0.844826	-3.10762	2.369095	O	-1.3558	2.845608	2.708549
C	2.992232	3.142254	-1.24166	C	-2.01488	-2.18298	-1.66527
C	1.622277	3.542819	-0.60766	C	-0.94568	-2.79727	-0.74039
C	0.415967	3.248345	-1.51019	C	0.388106	-3.0661	-1.45487
C	-0.96716	3.618002	-0.91404	C	1.451292	-3.81244	-0.61373
C	-2.11218	3.270974	-1.89464	C	2.725737	-4.07771	-1.46099
C	-3.52972	3.442906	-1.33326	C	3.970295	-4.44804	-0.65058
C	-4.59078	3.375817	-2.42884	C	5.061123	-5.05908	-1.51979
F	-1.97284	1.996673	-2.28919	F	3.024687	-3.00202	-2.20212
F	-2.02775	4.066773	-2.96788	F	2.452411	-5.09725	-2.28371
F	-4.39183	2.303196	-3.20971	F	5.383312	-4.16324	-2.47727
F	-5.78673	3.214585	-1.81593	F	6.119538	-5.28608	-0.75081
F	-3.78701	2.499118	-0.4224	F	4.44268	-3.33788	-0.05621
F	-3.6333	4.646416	-0.76015	F	3.63096	-5.33761	0.285846
F	-0.98297	4.941461	-0.71322	F	0.973557	-4.99975	-0.23368
F	0.53707	3.923221	-2.64885	F	0.159626	-3.76272	-2.56611
F	0.456707	1.922379	-1.77724	F	0.900803	-1.86644	-1.78815
F	1.472475	2.862095	0.53834	F	-0.70447	-1.91769	0.24613
F	1.635486	4.843612	-0.3414	F	-1.41173	-3.93395	-0.23728
F	3.839763	4.137508	-1.01386	F	-2.42983	-3.11918	-2.5086
F	2.856903	2.973506	-2.56373	F	-1.4548	-1.17885	-2.36568
O	-1.18782	2.893393	0.244185	O	1.793626	-3.00244	0.466372
C	-1.31118	3.508627	1.46672	C	1.496114	-3.32186	1.774446
C	-1.47644	2.373008	2.488047	C	1.664012	-2.03492	2.609003
C	-1.59686	2.886452	3.955727	C	1.707517	-2.34898	4.120182
O	-2.63396	1.746626	2.079151	O	2.831027	-1.45962	2.147431
C	-3.01571	0.532194	2.553688	C	3.207483	-0.18022	2.448051
C	-4.54742	0.517581	2.678412	C	4.544749	0.120302	1.767189
F	-0.24374	4.246957	1.735757	F	0.249259	-3.75679	1.888386

F	-2.38297	4.294834	1.508125	F	2.316741	-4.26039	2.226297
F	-0.40745	1.569629	2.391233	F	0.60158	-1.2489	2.379933
F	-1.19421	4.143266	4.051154	F	0.875414	-3.34186	4.393199
F	-2.85971	2.82624	4.345121	F	2.929045	-2.69904	4.472046
F	-0.85959	2.143725	4.753048	F	1.334023	-1.28762	4.807444
F	-2.65084	-0.42001	1.667493	F	2.292579	0.704268	2.010019
F	-2.44459	0.234476	3.712296	F	3.323165	-0.00181	3.763033
F	-4.89142	1.098353	3.824226	F	7.030684	-2.71427	-0.87894
F	-5.05281	1.276596	1.677819	F	5.107617	1.275209	-1.56457
H	9.418029	-2.47543	-1.4635	H	-9.99956	0.997765	-1.48104
H	-4.61315	4.293322	-3.03159	H	4.720852	-5.99187	-1.99171
S	-6.7773	-2.71146	-1.86042	S	6.498763	4.455248	-1.89206
O	-7.00799	-1.59099	-2.80317	O	7.732744	3.730508	-2.07018
O	-6.79468	-4.06947	-2.43499	O	5.932921	5.170857	-3.04065
O	-5.5155	-2.4338	-1.07917	O	5.414972	3.631385	-1.18586
O	-3.55411	-0.15237	-1.37032	O	3.17021	-0.69779	-0.47662
H	-2.04222	0.07061	-0.72328	H	1.793025	0.008856	-0.58972
H	-4.40516	-0.03575	-0.89776	H	3.139638	-1.48508	0.091909
H	-3.85512	-0.10631	-2.32358	H	3.863546	-0.89795	-1.14331
O	-4.69664	-0.41961	-3.69056	O	5.364393	-1.23985	-2.0281
H	-5.541	-0.90782	-3.57549	H	5.660015	-0.31474	-1.8512
H	-4.82934	0.208133	-4.41351	H	5.272518	-1.33518	-2.98947
O	-4.15959	-4.69203	-2.55346	O	3.409314	5.37735	-2.49267
H	-3.62354	-4.15149	-3.15573	H	2.7031	5.339431	-3.152
H	-5.09504	-4.61668	-2.84809	H	4.29554	5.399283	-2.94174
O	-3.74783	-2.9742	1.289825	O	3.326728	3.409185	1.183864
H	-2.7895	-2.83645	1.422604	H	2.410134	3.169832	1.430918
H	-4.2033	-2.21827	1.712105	H	3.883431	2.601675	1.352704
O	-2.52327	-5.17749	-0.03436	O	1.46326	5.047438	-0.47722
H	-2.21672	-5.86277	-0.64576	H	1.376768	5.984086	-0.24665
H	-1.74132	-4.69273	0.302423	H	0.887638	4.520935	0.119149
O	-5.63696	-5.17693	-0.13512	O	4.233955	5.847286	0.432482
H	-5.64316	-6.0177	0.342705	H	5.16473	6.021511	0.183712
H	-6.32678	-5.18693	-0.82975	H	4.251458	5.471299	1.329513
O	-2.65333	-2.61265	-1.48553	O	2.744505	2.490792	-1.59341

H	-1.8367	-2.58336	-0.9526	H	1.986004	1.982018	-1.23098
H	-3.01725	-1.6815	-1.47095	H	3.489688	1.858492	-1.68209
O	-7.9244	-2.69241	-0.77019	O	6.727482	5.579586	-0.72394
H	-7.66392	-2.06011	-0.04042	H	5.418604	2.199363	-1.34164
O	-5.01497	-0.73667	2.569558	O	4.81459	1.261879	1.462961
H	-5.91836	-0.74333	2.133526	H	7.530252	6.092394	-0.93463
Fe	-4.07496	-3.84557	-0.57756	Fe	3.392025	4.282747	-0.74016
O	-1.07888	-0.08304	-0.52085	O	0.957853	0.584382	-0.64572
H	-0.5515	0.490544	-1.09696	H	0.393926	0.181223	-1.32736
H	-0.47361	-0.49264	0.620038	H	0.143247	0.736575	0.569764
O	-6.22483	0.476769	-1.011	O	7.225523	-0.41551	0.120305
H	-6.53508	-0.03026	-1.79579	H	8.025461	0.06974	0.36627
H	-6.24535	1.42277	-1.23584	H	7.477915	-1.31557	-0.20447
O	-7.19607	-0.78866	1.045486	O	5.291352	-0.9093	1.620566
H	-6.86248	-0.16751	0.33048	H	6.333546	-2.2822	-1.39053
H	-8.0026	-0.38782	1.409673	H	6.141799	-0.65936	1.081775

Bond	Bond length (Å)
Dissociating C-S bond	6.000
Approaching O-C bond	1.343
Dissociating O-O bond	4.842

Fig. S3. Cartesian coordinates of the optimized geometries of doubly-hydrated Nafion membrane model plus monovalent Fe cation (Fe^{+}) hydration complex coordinating H_2O_2 for the geometrical change without and with the C-F bond dissociation of Nafion side chain with keeping the C-S distance at 6.0Å. The bond lengths of dissociating C-S, approaching O-C and dissociating O-O bonds, which are shown in Fig. 1, are also summarized in tables after the coordinate tables. The unit is Å.

(λ = 3)

Without C-F dissociation				With C-F dissociation			
Atom	X	Y	Z	Atom	X	Y	Z
C	9.191128	1.524179	-1.18038	C	-9.01687	1.947605	0.98609
C	7.924728	0.92113	-0.58864	C	-7.8059	1.18169	0.473417
C	6.638379	1.646276	-1.00085	C	-6.47603	1.92089	0.669474
C	5.421666	1.306402	-0.11036	C	-5.3439	1.394326	-0.24216
C	4.110044	1.795885	-0.77655	C	-3.96781	1.897003	0.262008
C	2.881414	1.832361	0.149513	C	-2.78104	1.704905	-0.70362
F	3.835608	0.9692	-1.78942	F	-3.67531	1.228002	1.380321
F	4.308625	3.029077	-1.25315	F	-4.07246	3.202919	0.541779
F	2.601045	0.575625	0.512782	F	-2.46827	0.407895	-0.69238
F	3.15966	2.566743	1.22581	F	-3.10451	2.084207	-1.93951
F	5.59698	1.906863	1.073273	F	-5.58144	1.847676	-1.48045
F	6.8619	2.961563	-0.91125	F	-6.66885	3.211964	0.378992
F	6.355198	1.328496	-2.26532	F	-6.11189	1.807052	1.94749
F	7.855264	-0.35535	-0.98935	F	-7.75585	0.01476	1.127725
F	8.039617	0.951955	0.746054	F	-7.99299	0.939903	-0.83152
F	9.476623	2.673914	-0.56338	F	-9.30174	2.955503	0.156669
F	8.996285	1.763316	-2.48481	F	-8.73896	2.443968	2.200239
O	5.3478	-0.07254	-0.00404	O	-5.3654	0.012497	-0.1624
C	5.28064	-0.74985	1.174982	C	-5.26676	-0.81083	-1.24509
C	4.736044	-2.13822	0.819214	C	-4.81254	-2.17554	-0.70308
C	4.388715	-3.00077	2.035079	C	-4.39824	-3.15479	-1.80588
O	3.563271	-1.97069	0.096448	O	-3.72028	-1.95569	0.115069
C	3.451753	-2.47086	-1.15947	C	-3.67811	-2.48096	1.369124
C	2.02161	-2.20269	-1.62244	C	-2.28129	-2.21882	1.922937

F	6.484338	-0.87639	1.7248	F	-6.44259	-0.94312	-1.84955
F	4.489096	-0.16072	2.069391	F	-4.39795	-0.35858	-2.14955
F	5.68443	-2.77313	0.115459	F	-5.85129	-2.70468	-0.03781
F	5.42793	-3.0729	2.853621	F	-5.35323	-3.22253	-2.72688
F	3.360479	-2.49045	2.683838	F	-3.2792	-2.7579	-2.37733
F	4.083025	-4.2142	1.616214	F	-4.22555	-4.35186	-1.28245
F	4.289675	-1.87782	-2.00977	F	-4.56865	-1.88498	2.166146
F	3.675076	-3.78061	-1.2077	F	-3.92613	-3.78565	1.386123
F	1.871224	-2.75738	-2.8221	F	-2.17676	-2.82802	3.100647
F	1.811517	-0.89387	-1.71204	F	-2.13703	-0.89988	2.10896
S	0.715567	-2.93031	-0.49627	S	-0.91658	-2.79641	0.806198
O	-0.27816	-3.47421	-1.43311	O	0.080452	-3.34254	1.801594
O	0.153648	-1.65246	0.193371	O	-0.42629	-1.53488	0.203544
O	1.339129	-3.78024	0.484859	O	-1.47627	-3.78897	-0.0943
C	1.624756	2.420562	-0.52335	C	-1.54187	2.524455	-0.27914
C	0.37628	2.366519	0.385085	C	-0.25704	2.097537	-1.01804
C	-0.86371	3.024375	-0.25038	C	1.001072	2.874738	-0.59069
C	-2.21756	2.871557	0.517238	C	2.318116	2.395098	-1.26616
C	-3.40152	3.040326	-0.45524	C	3.558948	2.951613	-0.53805
C	-4.78983	3.134604	0.181137	C	4.908102	2.549516	-1.14131
C	-5.85807	3.283192	-0.90339	C	6.088311	3.208972	-0.4368
F	-3.42612	1.979162	-1.27989	F	3.558455	2.50158	0.725145
F	-3.19049	4.146103	-1.18026	F	3.476877	4.282924	-0.52466
F	-7.05833	3.017986	-0.33572	F	7.187451	2.591637	-0.9092
F	-5.86227	4.55674	-1.31384	F	6.126183	4.493148	-0.81697
F	-5.01823	2.030844	0.893043	F	5.055011	1.204881	-1.0047
F	-4.82542	4.196431	0.994853	F	4.928089	2.835188	-2.44341
F	-2.25477	3.858241	1.424512	F	2.297161	2.863201	-2.5221
F	-0.62092	4.3219	-0.39241	F	0.832894	4.15866	-0.90426
F	-1.0007	2.48469	-1.48423	F	1.117907	2.764989	0.739625
F	0.115041	1.074701	0.623425	F	-0.05554	0.803459	-0.76761
F	0.642904	3.000568	1.523682	F	-0.44734	2.283671	-2.32544
F	1.853619	3.686924	-0.86153	F	-1.76178	3.813767	-0.51275
F	1.371954	1.699287	-1.62787	F	-1.36135	2.343431	1.046245
O	-2.35583	1.621918	1.095946	O	2.431125	1.010897	-1.21558

C	-2.3206	1.486094	2.455469	C	2.420166	0.256058	-2.34833
C	-2.44303	-0.00991	2.782925	C	2.687651	-1.21169	-1.91823
C	-2.49224	-0.25401	4.304664	C	2.025397	-2.22296	-2.86699
O	-3.58753	-0.40103	2.141644	O	4.062911	-1.27293	-1.85709
C	-4.13907	-1.65683	2.161115	C	4.81699	-2.40205	-1.75637
C	-3.27864	-2.81143	1.596978	C	6.243557	-1.94505	-1.42578
F	-1.1744	1.964246	2.938426	F	1.260044	0.36524	-2.99392
F	-3.31674	2.144953	3.041976	F	3.371382	0.63529	-3.20176
F	-1.34718	-0.63491	2.311087	F	2.155935	-1.44691	-0.70582
F	-1.55124	0.444938	4.919423	F	0.718602	-2.08197	-2.83782
F	-3.66801	0.102113	4.787553	F	2.470179	-2.04665	-4.09968
F	-2.28389	-1.53622	4.550672	F	2.313231	-3.45603	-2.46747
F	-4.54744	-1.98557	3.3842	F	4.354064	-3.21123	-0.77231
F	-5.21026	-1.54693	1.379659	F	4.809174	-3.09875	-2.8822
F	-2.54686	-2.29249	0.573181	F	6.169304	-1.54527	-0.08719
F	-2.43689	-3.2708	2.505647	F	3.733379	1.944153	3.35778
H	10.02077	0.810247	-1.05242	H	-9.87486	1.259434	1.052899
H	-5.70266	2.590746	-1.73726	H	6.037896	3.125393	0.660502
S	-7.12578	-0.01186	-2.05842	S	5.349558	1.788954	3.184854
O	-7.34049	0.590338	-3.36496	O	5.792285	1.247759	4.451128
O	-7.23375	-1.49061	-1.97508	O	5.788374	3.103294	2.749511
O	-5.8514	0.458089	-1.39963	O	5.365858	0.778592	2.061633
H	-0.90717	0.577846	-1.64721	H	-0.08303	1.061238	1.937653
O	-3.70906	-0.56681	-2.24007	O	3.009994	-0.21743	1.585663
H	-4.53262	-0.13027	-1.80721	H	3.799689	0.386947	1.592664
H	-3.84064	-0.43893	-3.19707	H	3.542639	-0.83563	3.931506
O	-2.88462	-4.40096	-1.3724	O	2.663079	-3.28506	1.506888
H	-1.91182	-4.32109	-1.39034	H	1.668262	-3.41363	1.471573
H	-3.1079	-4.57209	-0.44059	H	2.982322	-3.23719	0.591168
O	-3.35599	-2.30211	-4.22903	O	3.661159	-3.31416	4.112618
H	-2.35186	-2.23468	-4.21426	H	3.56006	-4.12905	3.590335
H	-3.60372	-2.9111	-4.93662	H	4.601688	-3.25003	4.349033
O	-5.52936	-3.12053	-0.9648	O	5.198257	-1.76635	2.396245
H	-5.96547	-3.95187	-1.21368	H	5.474407	-0.79736	2.389682
H	-6.21064	-2.40592	-1.23639	H	5.480491	-2.08572	1.521016

O	-0.83102	-2.02846	-3.76064	O	1.272282	-1.66529	3.470887
H	-0.4599	-2.77812	-3.26547	H	0.703328	-2.35303	3.038304
H	-0.8456	-1.31639	-3.08603	H	0.868207	-0.8111	3.173003
O	-8.28024	0.495941	-1.06165	O	6.651193	-0.89426	-2.15503
H	-8.19739	1.461527	-0.98752	H	6.138459	-0.10166	-1.90748
O	-4.05745	-3.81012	1.156834	O	7.068676	-2.98773	-1.55626
H	-4.72421	-3.45886	0.495738	H	7.963639	-2.64255	-1.70913
Fe	-3.82048	-2.72066	-2.20676	Fe	3.181756	-1.75224	2.802649
O	-1.1947	-0.34652	-1.58139	O	0.481034	0.275528	1.891785
H	-2.19117	-0.33691	-1.72336	H	2.14401	0.268917	1.602935
H	-0.50613	-1.16919	-0.41909	H	0.078135	-0.32938	1.202472

Bond	Bond length (Å)
Dissociating C-S bond	6.000
Approaching O-C bond	1.341
Dissociating O-O bond	6.426

(λ = 4)

	Without C-F dissociation				With C-F dissociation		
Atom	X	Y	Z	Atom	X	Y	Z
C	-8.53058	0.888493	-2.72839	C	-8.19692	0.813915	-2.64625
C	-7.45178	0.565127	-1.70507	C	-7.1377	0.225743	-1.72339
C	-6.07389	0.292219	-2.32346	C	-5.75344	0.083428	-2.37026
C	-5.13231	-0.49846	-1.38532	C	-4.74767	-0.73812	-1.53797
C	-3.66979	-0.41481	-1.89	C	-3.35305	-0.80091	-2.20283
C	-2.65607	-1.36842	-1.22625	C	-2.31341	-1.60454	-1.39963
F	-3.23062	0.828636	-1.69194	F	-2.87905	0.454306	-2.32364
F	-3.67228	-0.67608	-3.20579	F	-3.4634	-1.33921	-3.41713
F	-2.35261	-0.89265	-0.01762	F	-1.98901	-0.88481	-0.32355
F	-3.17141	-2.59291	-1.11615	F	-2.83101	-2.77126	-1.01825
F	-5.54813	-1.7729	-1.38843	F	-5.20636	-1.99273	-1.42974
F	-6.2502	-0.43226	-3.43389	F	-5.90921	-0.51589	-3.55589
F	-5.51578	1.459687	-2.64597	F	-5.25059	1.305655	-2.55461

F	-7.35581	1.608484	-0.8722	F	-7.03464	1.029553	-0.65439
F	-7.85176	-0.50943	-1.01096	F	-7.57569	-0.97428	-1.32482
F	-8.89185	-0.22362	-3.37497	F	-8.5303	-0.08635	-3.57605
F	-8.04459	1.771588	-3.61278	F	-7.7126	1.906297	-3.25057
O	-5.21632	0.092804	-0.13784	O	-4.59783	-0.09548	-0.31744
C	-5.30963	-0.61425	1.027108	C	-4.88247	-0.70576	0.868293
C	-4.81515	0.322296	2.14001	C	-4.74563	0.35304	1.97898
C	-4.61707	-0.38902	3.482605	C	-4.61735	-0.31732	3.363048
O	-3.60353	0.826997	1.72024	O	-3.6288	1.105868	1.680185
C	-3.33817	2.156362	1.781481	C	-3.55448	2.413358	2.064271
C	-1.88297	2.346032	1.373836	C	-2.1448	2.912822	1.763186
F	-6.56648	-0.97176	1.263037	F	-6.12419	-1.18164	0.882351
F	-4.5698	-1.72354	0.999864	F	-4.05259	-1.72525	1.097482
F	-5.74298	1.278574	2.307194	F	-5.86632	1.092079	1.972172
F	-5.72818	-1.04511	3.804006	F	-5.40639	-1.38692	3.412456
F	-3.61972	-1.24542	3.412407	F	-3.37265	-0.69027	3.570883
F	-4.36697	0.505933	4.416958	F	-4.99617	0.512539	4.31522
F	-4.10922	2.835945	0.925264	F	-4.41469	3.163587	1.373299
F	-3.5377	2.666455	2.993451	F	-3.82448	2.572298	3.354398
F	-1.66174	3.665319	1.281347	F	-2.01386	4.118621	2.30699
F	-1.71734	1.813376	0.156361	F	-2.01038	3.023845	0.432041
S	-0.64189	1.577941	2.535736	S	-0.78653	1.831274	2.371158
O	-0.00199	2.761909	3.176835	O	0.366417	2.807382	2.324444
O	0.275159	0.892754	1.586691	O	-0.64329	0.805109	1.310125
O	-1.39526	0.707419	3.424384	O	-1.17318	1.370892	3.691559
C	-1.35403	-1.50406	-2.04466	C	-1.04132	-1.92184	-2.21939
C	-0.20198	-2.09404	-1.20591	C	0.139578	-2.32707	-1.32246
C	1.041325	-2.50181	-2.02615	C	1.42797	-2.68932	-2.09018
C	2.011279	-3.38683	-1.20622	C	2.421346	-3.48855	-1.20245
C	3.414031	-3.52796	-1.85014	C	3.89204	-3.36756	-1.66502
C	4.463017	-2.51418	-1.3955	C	4.694138	-2.21661	-1.04874
C	5.702131	-2.51775	-2.2831	C	5.991251	-1.9463	-1.80266
F	3.296507	-3.44482	-3.18533	F	3.922485	-3.2512	-3.00245
F	3.897054	-4.73753	-1.55228	F	4.531508	-4.49308	-1.33549
F	5.393541	-1.89882	-3.4494	F	5.673125	-1.29355	-2.94964

F	6.643673	-1.8024	-1.67273	F	6.739597	-1.14005	-1.05804
F	3.929977	-1.28004	-1.42897	F	3.965633	-1.08341	-1.06782
F	4.841308	-2.79352	-0.15108	F	4.989887	-2.51454	0.214509
F	1.482613	-4.61892	-1.1748	F	2.086169	-4.78101	-1.30421
F	0.684013	-3.20431	-3.09906	F	1.150162	-3.43509	-3.15576
F	1.642531	-1.37316	-2.42826	F	1.979627	-1.54156	-2.50811
F	0.17195	-1.15535	-0.33292	F	0.438396	-1.30177	-0.52028
F	-0.65229	-3.16685	-0.54965	F	-0.24826	-3.37903	-0.5934
F	-1.60349	-2.26381	-3.10461	F	-1.33168	-2.92172	-3.04876
F	-0.95128	-0.28565	-2.46646	F	-0.67317	-0.84823	-2.93377
O	2.106399	-2.82643	0.049984	O	2.295948	-3.02142	0.091928
C	2.156616	-3.58337	1.187983	C	2.328117	-3.85277	1.178338
C	2.28097	-2.57906	2.339806	C	2.134017	-2.94185	2.396105
C	2.394538	-3.24096	3.714189	C	2.198883	-3.68376	3.732451
O	3.436449	-1.83611	2.143778	O	3.154494	-2.00163	2.396387
C	3.355419	-0.48522	2.011922	C	2.865087	-0.66719	2.318225
C	4.765815	0.114006	1.854475	C	4.212253	0.046339	2.352323
F	1.045492	-4.29434	1.344948	F	1.347037	-4.74626	1.141321
F	3.186101	-4.42757	1.155535	F	3.484704	-4.50899	1.241941
F	1.169771	-1.83458	2.326317	F	0.919081	-2.38956	2.286111
F	1.326048	-3.97836	3.962271	F	1.266927	-4.61962	3.785612
F	3.47029	-4.01035	3.763584	F	3.385956	-4.24587	3.885468
F	2.49465	-2.29932	4.633187	F	1.999974	-2.81682	4.70645
F	2.649363	-0.16042	0.911398	F	2.199828	-0.40476	1.179157
F	2.7524	0.071338	3.059759	F	2.110028	-0.26815	3.328319
F	5.650179	-0.62512	2.53189	F	4.711689	0.158235	1.129262
F	5.112431	-0.03242	0.524799	F	4.220278	6.512168	-1.01109
H	-9.39996	1.322482	-2.2088	H	-9.08054	1.080696	-2.04399
H	6.062992	-3.53342	-2.50047	H	6.537852	-2.86651	-2.05496
S	5.084946	3.792199	-2.8715	S	4.960381	3.722817	-2.32997
O	5.674722	2.558946	-3.45047	O	5.362721	2.736596	-3.37677
O	3.629193	3.900694	-2.98572	O	5.326035	5.114484	-2.67067
O	5.600428	4.051664	-1.49646	O	3.524401	3.566401	-1.98599
O	3.358529	1.307097	-1.29913	O	2.247139	1.049892	-1.22986
H	2.3436	1.315392	-1.10302	H	1.257865	1.109537	-1.37003

H	3.809556	0.785637	-0.61068	H	2.375575	0.382289	-0.53638
H	3.631654	0.925656	-2.34445	H	3.173077	0.967929	-2.39626
O	4.015885	0.631125	-3.50271	O	3.840163	0.850786	-3.1834
H	4.700385	1.357131	-3.66567	H	4.522248	1.711202	-3.31
H	4.470142	-0.22641	-3.53768	H	4.371927	0.049716	-3.02936
O	4.430887	5.051065	0.604267	O	1.747028	6.161732	0.131525
H	4.407337	6.005428	0.760121	H	1.316113	5.388123	-0.28605
H	4.775396	4.8926	-0.31443	H	2.500689	6.390133	-0.44254
O	2.680815	3.095747	2.755567	O	2.868158	2.123599	1.897174
H	1.80935	2.800319	3.097592	H	1.910425	2.225599	2.146622
H	3.305175	2.350462	2.807579	H	3.334422	2.714252	2.510964
O	0.692936	4.982712	1.806287	O	2.440254	4.975709	2.390895
H	0.014145	5.182591	1.147765	H	2.109857	5.534017	1.629638
H	0.287053	4.310014	2.398581	H	1.672215	4.462883	2.691335
O	6.114126	3.006575	0.964348	O	4.419187	4.542992	0.759175
H	5.641577	3.843367	1.174934	H	3.842543	4.668275	1.56368
H	6.112868	3.025605	-0.01665	H	4.346464	5.386681	0.256403
O	1.841153	4.0765	-0.97686	O	0.824149	3.651733	-0.25616
H	1.302379	3.267219	-1.07935	H	0.519971	3.510782	0.6666
H	2.493059	4.054824	-1.71497	H	0.275604	3.040043	-0.79382
O	5.64695	4.9913	-3.78504	O	5.801821	3.280682	-1.05804
H	6.570963	4.78299	-4.00574	H	4.668415	6.043602	-1.76981
O	4.754647	1.37897	2.191685	O	4.782666	0.369032	3.323138
H	5.415	2.000964	1.63372	H	5.425231	3.718827	-0.2348
Fe	2.471126	4.108901	0.995147	Fe	2.929424	3.019701	-0.0579
O	0.900932	1.488244	-0.8304	O	-0.32402	1.41066	-1.20549
H	0.245916	1.002571	-1.3555	H	-1.086	1.209329	-1.7669
H	0.666836	1.286765	0.134604	H	-0.55572	1.132803	-0.27187

Bond	Bond length (Å)
Dissociating C-S bond	6.000
Approaching O-C bond	1.309
Dissociating O-O bond	7.040

(λ = 5)

	Without C-F dissociation				With C-F dissociation		
Atom	X	Y	Z	Atom	X	Y	Z
C	-9.07791	1.023977	-1.61223	C	-8.08517	0.984306	-3.22161
C	-7.85304	0.631828	-0.79714	C	-7.16115	0.46011	-2.12981
C	-6.65621	0.18594	-1.64895	C	-5.73439	0.1259	-2.63152
C	-5.52292	-0.49682	-0.85836	C	-4.86169	-0.72538	-1.70401
C	-4.42102	-1.03825	-1.80983	C	-3.37858	-0.68469	-2.13384
C	-3.11298	-1.40402	-1.09446	C	-2.47587	-1.62391	-1.31309
F	-4.12441	-0.13807	-2.75029	F	-2.90627	0.577089	-1.97046
F	-4.91363	-2.13469	-2.39183	F	-3.28598	-0.99937	-3.42069
F	-2.58646	-0.29	-0.57862	F	-2.31829	-0.98995	-0.13972
F	-3.39239	-2.27477	-0.12062	F	-3.03081	-2.81236	-1.12215
F	-6.00706	-1.55711	-0.194	F	-5.25543	-2.00657	-1.79397
F	-7.09217	-0.66563	-2.58563	F	-5.86999	-0.51694	-3.79173
F	-6.15646	1.273843	-2.23789	F	-5.07578	1.270003	-2.8388
F	-7.48176	1.701521	-0.07712	F	-7.09322	1.413402	-1.18127
F	-8.22438	-0.34475	0.038263	F	-7.75211	-0.62444	-1.62764
F	-9.64718	-0.0732	-2.12148	F	-8.44191	-0.02101	-4.02748
F	-8.71255	1.831573	-2.61614	F	-7.45297	1.923139	-3.94094
O	-4.99553	0.4628	-0.00877	O	-4.98378	-0.19816	-0.4272
C	-5.00236	0.315172	1.347062	C	-5.26253	-0.96646	0.665479
C	-4.35797	1.584216	1.914418	C	-5.21059	-0.02883	1.875813
C	-4.10218	1.516123	3.423565	C	-5.11692	-0.80707	3.173313
O	-3.13762	1.737057	1.282127	O	-4.09484	0.77853	1.757006
C	-2.80436	2.921173	0.704942	C	-4.2273	2.12304	1.931369
C	-1.36177	2.80274	0.223415	C	-2.83102	2.71335	1.813749
F	-6.24161	0.189964	1.817652	F	-6.49059	-1.47562	0.640941
F	-4.31055	-0.75511	1.733776	F	-4.40988	-1.97993	0.819207
F	-5.1971	2.603174	1.674694	F	-6.35966	0.661286	1.876063
F	-5.2218	1.18866	4.057334	F	-6.03115	-1.77139	3.148857
F	-3.17621	0.618585	3.694107	F	-3.92023	-1.34567	3.289639
F	-3.70287	2.699343	3.846395	F	-5.35818	0.017382	4.166257
F	-3.58296	3.178554	-0.3491	F	-5.01205	2.651899	0.988332

F	-2.90964	3.942029	1.547573	F	-4.71228	2.435325	3.125367
F	-0.95034	4.020926	-0.12716	F	-2.8827	3.967999	2.241863
F	-1.32616	2.014702	-0.86166	F	-2.47451	2.733032	0.519586
S	-0.18245	2.12153	1.477859	S	-1.53859	1.758879	2.720304
O	1.076323	2.843281	1.039865	O	-0.5118	2.858805	2.935727
O	-0.08567	0.678442	1.170533	O	-1.12407	0.803227	1.666258
O	-0.69088	2.510403	2.781336	O	-2.08441	1.209854	3.941496
C	-2.03703	-2.03099	-2.00544	C	-1.14079	-1.8975	-2.04188
C	-0.87668	-2.60872	-1.1761	C	-0.04194	-2.53166	-1.14783
C	0.354347	-3.0002	-2.01191	C	1.354859	-2.40859	-1.76972
C	1.391198	-3.79441	-1.17809	C	2.452307	-3.17909	-0.99392
C	2.802929	-3.80092	-1.80729	C	3.853825	-2.99325	-1.6197
C	3.764973	-2.66315	-1.44799	C	5.005912	-3.51119	-0.77641
C	5.090501	-2.8139	-2.18449	C	6.271969	-3.61165	-1.60931
F	2.676238	-3.85549	-3.1402	F	4.10603	-1.7121	-1.85639
F	3.41932	-4.92139	-1.40098	F	3.876546	-3.671	-2.78695
F	4.911823	-2.57725	-3.4993	F	6.58975	-2.39359	-2.11035
F	5.918678	-1.86718	-1.71181	F	7.269236	-4.00493	-0.81541
F	3.248056	-1.46227	-1.72438	F	5.206596	-2.69021	0.256972
F	4.046732	-2.72011	-0.13911	F	4.704858	-4.7343	-0.31413
F	0.987643	-5.07224	-1.15367	F	2.180206	-4.49331	-1.00061
F	-0.04458	-3.77377	-3.02304	F	1.310239	-2.82028	-3.04288
F	0.919901	-1.89954	-2.51207	F	1.665892	-1.10778	-1.72753
F	-0.52896	-1.68148	-0.28262	F	0.029365	-1.95511	0.044035
F	-1.29479	-3.71192	-0.54748	F	-0.38401	-3.81671	-0.99222
F	-2.5569	-2.991	-2.76338	F	-1.41659	-2.76056	-3.0222
F	-1.54318	-1.06098	-2.7947	F	-0.70072	-0.76984	-2.60205
O	1.432713	-3.2357	0.083263	O	2.48263	-2.63757	0.269572
C	1.465733	-3.99284	1.219935	C	2.182755	-3.33848	1.389794
C	1.449172	-2.99485	2.386787	C	1.872386	-2.3237	2.514652
C	1.142755	-3.66411	3.731	C	2.164429	-2.93577	3.889577
O	2.712004	-2.43495	2.46527	O	2.662338	-1.19929	2.441491
C	2.87355	-1.08605	2.490395	C	2.254063	-0.03796	1.8379
C	4.324199	-0.78585	2.870579	C	3.504046	0.700096	1.369156
F	0.399998	-4.78569	1.310736	F	1.102325	-4.11229	1.238308

F	2.554026	-4.75654	1.25145	F	3.213067	-4.11284	1.729208
F	0.492339	-2.09284	2.143036	F	0.560395	-2.03996	2.474372
F	-0.12638	-4.02102	3.793271	F	1.607129	-4.13693	3.979478
F	1.904281	-4.73858	3.881347	F	3.462964	-3.03517	4.104247
F	1.399293	-2.81517	4.709089	F	1.639794	-2.15607	4.815346
F	2.63736	-0.56003	1.272023	F	1.440024	-0.24079	0.79458
F	2.062389	-0.48708	3.353593	F	1.574729	0.707052	2.721407
F	4.586937	-1.29797	4.064974	F	3.455465	0.997455	-1.94013
F	5.11061	-1.45714	1.970559	F	5.936733	2.643356	1.208637
H	-9.7892	1.546919	-0.95251	H	-8.98177	1.421063	-2.75245
H	5.533303	-3.8092	-2.04343	H	6.147789	-4.31576	-2.44236
S	5.98571	2.889581	-1.57134	S	5.750146	4.309594	-2.86481
O	6.428031	1.493696	-1.95129	O	6.949078	4.297612	-1.97249
O	6.227184	3.87477	-2.62996	O	4.647158	5.02493	-2.0761
O	4.467228	2.794704	-1.26403	O	3.088167	1.697271	0.391812
O	2.927426	1.367002	-2.54767	O	1.409285	1.852142	-2.62305
H	2.240221	0.893099	-1.94288	H	0.743262	1.519556	-1.96127
H	3.579629	1.984334	-1.98968	H	2.407068	1.353029	-2.38403
H	3.582807	0.753356	-3.07557	H	1.582202	2.798674	-2.3806
O	4.812637	0.258455	-3.67688	O	5.916169	0.382915	-1.40389
H	5.560525	0.657602	-3.15931	H	7.005842	1.517993	-1.10986
H	5.018102	-0.66801	-3.85818	H	6.176961	-0.52041	-1.6488
O	4.491794	5.826257	-2.14584	O	2.285112	4.121095	-1.62456
H	4.143459	6.283218	-2.92074	H	1.772327	4.939732	-1.51279
H	5.20844	5.220191	-2.45644	H	3.191821	4.415772	-1.97305
O	3.668626	2.199479	1.237385	O	2.3676	3.389583	2.686346
H	2.69285	2.385458	1.231696	H	1.454145	3.100543	2.933512
H	3.971356	2.333971	0.30089	H	2.968262	2.696274	2.992255
O	1.79935	5.45224	0.776983	O	2.251129	5.888818	0.763055
H	2.316138	5.716603	1.551974	H	3.147487	6.112674	1.069393
H	1.315119	4.639093	1.030913	H	1.648638	6.077361	1.499279
O	4.61813	4.856998	0.94577	O	4.41726	4.454679	0.523075
H	4.344527	4.004482	1.334397	H	4.693616	4.766462	-0.36691
H	5.499013	4.65707	0.574741	H	5.116919	3.780588	0.849425
O	1.600835	3.725905	-1.56508	O	0.482048	3.60158	0.385504

H	1.230872	3.231097	-0.80916	H	0.046133	3.454064	1.294444
H	1.789548	3.078389	-2.25956	H	0.108579	2.881456	-0.15597
O	6.615328	3.280042	-0.25219	O	5.346253	3.019458	-3.47
H	6.118016	1.509794	2.240225	H	3.173058	1.282147	-0.54159
O	4.600563	0.516655	2.847955	O	4.066823	1.365269	2.379113
H	4.092641	1.070857	2.147373	H	4.897563	1.824499	2.025334
Fe	3.170657	4.948212	-0.76981	Fe	2.516647	3.69278	0.583797
O	1.065324	0.451345	-1.16543	O	-0.43708	1.344628	-0.79609
H	0.225214	0.364994	-1.63931	H	-1.34747	1.390435	-1.12788
H	0.800152	0.429055	-0.20492	H	-0.55046	1.007674	0.137626
O	6.945947	-0.14108	0.047118	O	7.451003	2.229751	-0.56292
H	6.771863	0.373329	-0.79582	H	7.380107	3.077713	-1.11775
H	6.236978	-0.79232	0.165181	H	6.823531	2.430185	0.317717
O	6.882194	1.666322	1.644215	O	4.237803	-0.29177	0.800536
H	6.968364	0.776576	1.007915	H	5.070791	0.674138	-1.82379
H	6.71943	2.413368	0.936041	H	4.918413	0.026804	0.174141

Bond	Bond length (Å)
Dissociating C-S bond	6.000
Approaching O-C bond	1.332
Dissociating O-O bond	4.616
